# Evolutionary Conservation and Emerging Functional Diversity of the Cytosolic Hsp70:J Protein Chaperone Network of *Arabidopsis thaliana*

**DOI:** 10.1534/g3.117.042291

**Published:** 2017-04-21

**Authors:** Amit K. Verma, Danish Diwan, Sandeep Raut, Neha Dobriyal, Rebecca E. Brown, Vinita Gowda, Justin K. Hines, Chandan Sahi

**Affiliations:** * Department of Biological Sciences, Indian Institute of Science Education and Research, Bhopal, Madhya Pradesh 462066, India; † Department of Chemistry, Lafayette College, Easton, Pennsylvania 18042-1768

**Keywords:** *Arabidopsis thaliana*, Hsp40, Hsp70, J protein, yeast, evolution

## Abstract

Heat shock proteins of 70 kDa (Hsp70s) partner with structurally diverse Hsp40s (J proteins), generating distinct chaperone networks in various cellular compartments that perform myriad housekeeping and stress-associated functions in all organisms. Plants, being sessile, need to constantly maintain their cellular proteostasis in response to external environmental cues. In these situations, the Hsp70:J protein machines may play an important role in fine-tuning cellular protein quality control. Although ubiquitous, the functional specificity and complexity of the plant Hsp70:J protein network has not been studied. Here, we analyzed the J protein network in the cytosol of *Arabidopsis thaliana* and, using yeast genetics, show that the functional specificities of most plant J proteins in fundamental chaperone functions are conserved across long evolutionary timescales. Detailed phylogenetic and functional analysis revealed that increased number, regulatory differences, and neofunctionalization in J proteins together contribute to the emerging functional diversity and complexity in the Hsp70:J protein network in higher plants. Based on the data presented, we propose that higher plants have orchestrated their “chaperome,” especially their J protein complement, according to their specialized cellular and physiological stipulations.

Being sessile, plants have to deal with complex environmental cues including a variety of stresses. They have evolved with specific mechanisms that help them regulate their cellular proteome with the changing external environment (Kosova *et al.* 2011; Kurepa *et al.* 2009). Molecular chaperones are a diverse group of proteins that play critical roles in maintaining cellular proteostasis in all organisms, including plants, under normal as well as stress conditions (Boston *et al.* 1996; Bukau *et al.* 2006; Hartl *et al.* 2011; Miernyk 1999; Wang *et al.* 2004). The Hsp70 class of molecular chaperones is a large and evolutionary conserved family of proteins known to perform myriad cellular functions (Bukau and Horwich 1998; Kampinga and Craig 2010). Hsp70s never work alone. They always partner with multiple, structurally diverse J proteins (Hsp40s) to constitute the Hsp70:J protein chaperone network (Kampinga and Craig 2010). J proteins interact with Hsp70s through their conserved and signature J domain and stimulate their otherwise weak intrinsic ATPase activity. ATP hydrolysis results in profound conformational changes in the client binding domain (CBD), thereby modulating substrate executions and thus driving Hsp70’s functions (Kampinga and Craig 2010). In this way, Hsp70s, along with their obligate cochaperones, the J proteins, form a formidable chaperone network that performs various protein folding, remodelling, and quality control functions.

The J domain, the defining feature of all J proteins, is a compact tetrahelical domain of ∼70 aa residues with a highly conserved and functionally critical histidine, proline, and aspartic acid (HPD) tripeptide motif. Although J domains are critical for J protein function, often the regions outside the J domain determine the interaction of J proteins with their clients or affect their subcellular localization and thereby dictate the functional specificity of a J protein (Sahi and Craig 2007). Historically, J proteins have been classified into three classes based on similarity to the *Escherichia coli* protein DnaJ. Class I J proteins have domain organization similar to DnaJ, possessing an N-terminal J domain followed by a glycine/phenylalanine (G/F)-rich region, four repeats of the CxxCxGxG-type zinc finger, and a C-terminal CBD. Class II J proteins are very similar to class I, except that they lack the CxxCxGxG-type zinc finger. The CBD found in class I and class II J proteins has a characteristic hydrophobic pocket known to bind short hydrophobic patches on client proteins, thus determining their substrate specificity (Cheetham and Caplan 1998). All other J proteins that do not fit into either class I or class II are arbitrarily placed in class III. Thus, class III J proteins are structurally and functionally the most diverse, sharing only a J domain (Walsh *et al.* 2004).

Compared to other cellular compartments, the cytoplasm is the hub for most diverse cellular processes requiring many different chaperones. Consistent with this, the Hsp70:J protein network is most complex in the cytosol. For instance, in the case of *Saccharomyces cerevisiae*, out of a total of 22 J proteins, 13 are cytosolic, and most of these work with the same class of Hsp70, Ssa, generating a complex chaperone network that oversees different processes in the yeast cytosol (Sahi and Craig 2007). The number of J proteins has dramatically increased in plants (Cheetham and Caplan 1998; Nover and Miernyk 2001; Rajan and D’Silva 2009; Sarkar *et al.* 2013), which underlines the requirement of highly complex and possibly combinatorial Hsp70:J protein networks in plants. Although some plant J proteins have been shown to be associated with signaling (Bekh-Ochir *et al.* 2013), ion transport (Yang *et al.* 2010), photosynthesis (Kong *et al.* 2014; Wang *et al.* 2015), β-carotene synthesis (Lu *et al.* 2006), Fe–S cluster biogenesis (Dorn *et al.* 2010), and male sterility (Yang *et al.* 2009), the molecular mechanisms underlying their function as a J protein is largely elusive. We attempted to dissect the J protein network in *Arabidopsis thaliana* by implementing the phylogenetic computational amendments supplemented with functional assessment using *S. cerevisiae* as a genetic tool. We report that even though the functional specificities of most cytosolic J proteins are evolutionarily maintained, the Hsp70:J protein network in the *A. thaliana* cytosol is incredibly complex. Our work shows that this increased complexity cannot be accounted for alone by the higher number of J proteins in plants. Based on the data presented we propose that, besides the emergence of novel J proteins, regulatory differences and neofunctionalizations are together contributing to the functional diversity and emerging complexity of the Hsp70:J protein network in higher plants.

## Materials and Methods

### Ortholog detection and gene-tree construction

J proteins in *A. thaliana* were identified by WU-BLAST2 searches in the TAIR (https://www.arabidopsis.org/wublast/index2.jsp) database using each of the 22 J proteins of *S. cerevisiae* as a query sequence. The top results with significant high score and low *E*-value were shortlisted and used to perform the “reverse BLAST” in the *Saccharomyces* Genome Database (http://www.yeastgenome.org/). Sequences that resulted in the same J protein as was used in the query search were considered orthologous (Gabaldon and Koonin 2013). Cwc23 orthologs were separately identified using the Cwf23 sequence (Cwc23 ortholog in *Schizosaccharomyces pombe*) lacking the J domain as described previously (Sahi *et al.* 2010). Different domains and motifs were predicted using the SMART database (http://smart.embl-heidelberg.de/). Secondary structures were predicted using SWISS-MODEL (Arnold *et al.* 2006). Subcellular localization was assigned using the SUBAcon, WoLFPSORT, TargetP, and MultiLoc servers. Orthologous J protein sequences were aligned using MAFFT version7 (http://mafft.cbrc.jp/alignment/server/) using a “slow, progressive method” (Katoh *et al.* 2002). A phylogenetic tree was constructed using the approximate maximum likelihood method using FastTree at the ETE3 phylogenetic pipeline v3.0.0b32 on www.genome.jp (Huerta-Cepas *et al.* 2016). Sequence similarity was calculated by taking the arithmetic mean of the branch lengths of all the terminals and averaging them across a monophyletic clade. A *t*-test was performed under the nonparametric model using the Mann–Whitney *U*-test (GraphPad Prism v. 5.00) to identify clade-wise differences in branch length.

### Plasmid construction

Open reading frames (ORFs) corresponding to full-length or J domain-containing fragments of cytosolic J protein orthologs were PCR amplified from a pooled *Arabidopsis* cDNA sample made from RNA isolated from stressed and unstressed shoots, roots, and inflorescences. Yeast genes were amplified from yeast genomic DNA. J protein-encoding genes were cloned either into *HIS3*-marked pRS413 or *TRP1*-marked pRS414 yeast expression vectors under the *TEF* promoter (Mumberg *et al.* 1995) using standard methods (Table 1). To generate N-terminus HA-tagged constructs, the HA-tag-encoding sequence was added in the forward primers before the ORF. All the constructs were confirmed by restriction digestion and sequencing. All the primers used in the study are listed in Supplemental Material, Table S1 in File S1.

**Table 1:** List of plasmid constructs used in study

Plasmid	Gene	Base Vector	Source
pCS65	Ydj1	pRS413-*TEF*	This study
pCS65	Ydj1_1–220_	pRS413-*TEF*	This study
pCS20-A	atDjA2	pRS413-*TEF*	This study
pCS20-B	atDjA2	pRS414-*TEF*	This study
pCS21-A	atDjA1	pRS413-*TEF*	This study
pCS21-B	atDjA1	pRS414-*TEF*	This study
pCS436	atDjA2*_H42Q_*	pRS413-*TEF*	This study
pCS437	atDjA1*_H42Q_*	pRS413-*TEF*	This study
pCS435-A	atDjA2_1–128_	pRS413-*TEF*	This study
pCS435-B	atDjA1_1–127_	pRS413-*TEF*	This study
pCS662	atDjA2_HA	pRS413-*TEF*	This study
pCS663	atDjA1_HA	pRS413-*TEF*	This study
pCS26-A	atDjC10	pRS413-*TEF*	This study
pCS26-B	atDjC10	pRS414-*TEF*	This study
pCS507	atDjC10*_H486Q_*	pRS413-*TEF*	This study
pCS664	atDjC10_HA	pRS413-*TEF*	This study
pCS46	Jjj3	pRS413-*TEF*	This study
pCS336	atDjC13	pRS413-*TEF*	This study
pCS28	atDjC13	pRS414-*TEF*	This study
pCS253	atDjC13*_H39Q_*	pRS413-*TEF*	This study
pCS666	atDjC13_HA	pRS413-*TEF*	This study
pCS457	Sis1	pRS414-*TEF*	This study
pCS517	atDjB1	pRS413-*TEF*	This study
pCS24	atDjB1	pRS414-*TEF*	This study
pCS384	atDjB1*_H32Q_*	pRS414-*TEF*	This study
pCS517-B	atDjB1_HA	pRS413-*TEF*	This study
pCS27-A	atDjC12	pRS413-*TEF*	This study
pCS27-B	atDjC12	pRS414-*TEF*	This study
pCS665	atDjC12_HA	pRS413-*TEF*	This study
pCS19	atDjC37	pRS414-*TEF*	This study
pCS57	Cwc23	pRS414-*TEF*	This study
pCS667	atDjC37_HA	pRS413-*TEF*	This study
pCS141	Mdj1_1–186_	pRS414-*TEF*	This study
pCS76	Mdj1_56–186_	pRS414-*TEF*	This study
Xdj1	*XDJ1*-Xdj1	pRS313	Sahi *et al.* (2013)
Apj1	*APJ1*-Apj1	pRS314	Sahi *et al.* (2013)
Ydj1	*YDJ1*-Ydj1	pRS314	Sahi and Craig (2007)
Sis1_1–121_	*SIS1*-Sis1_1–121_	pRS313	Johnson and Craig (2001)
Sis1	*SIS1*-Sis1	pRS313	Yan and Craig (1999)
Sis1	*SIS1*-Sis1	pRS316	Yan and Craig (1999)
Rnq1-GFP	CUP1-Rnq1-GFP	pRS416	Lopez *et al.* (2003)

### Yeast methods

Yeast deletion strains (Table 2) were transformed with plasmids expressing specific J proteins following the standard LiAc/PEG method. The ability of the Sis1 and Cwc23 orthologs in *A. thaliana* to rescue the essential functions of Sis1 or Cwc23 was tested by the 5-FOA counterselection method. To test Jjj3 function, the *jjj3*Δ strain was double transformed with a *URA3*-marked *GAL*-DT plasmid (Mattheakis *et al.* 1992) and *HIS3*-marked plasmids expressing different J proteins, and their growth was accessed on galactose-containing plates. Plasmid shuffling experiments for prion maintenance were performed using strains bearing [*PSI*^+^]^Sc4^, [*PSI*^+^]^Sc37^, [*RNQ*^+^]^STR^, or [*URE3-1*] as described previously (Harris *et al.* 2014; Troisi *et al.* 2015). [*RNQ*^+^] aggregates in cells were observed directly under a fluorescent microscope following transformation by (pRS416-*CUP1*-Rnq1-GFP) plasmid (Aron *et al.* 2007). To create [*prion*^−^] control strains, prion-bearing cells were treated with the Hsp104 inhibitor guanidine hydrochloride (4 mM) and grown in liquid culture for 2 d at 30°.

**Table 2:** Yeast strains used in the study

Yeast Strain	Genotype	Reference
BY4743	*MATa/*α *his3*Δ*1/his3*Δ*1 leu2*Δ*0/leu2*Δ*0 LYS2/lys2*Δ*0 met15*Δ*0/MET15 ura3*Δ*0/ura3*Δ*0*	Thermo yeast knockout collection
W303	*MAT*α*leu2-3*,*112 trp1-1 can1-100 ura3-1 ade2-1 his3-11*,*15* [*phi^+^*]	Thomas and Rothstein (1989)
*ydj1*Δ	*MAT*α (*leu2-3*,*112 trp1-1 can1-100 ura3-1 ade2-1 his3-11*,*15*) [*phi^+^*] *ydj1*::*LEU2*	Sahi and Craig (2007)
*jjj3*Δ	*MATa*(*leu2-3*,*112 trp1-1 can1-100 ura3-1 ade2-1 his3-11*,*15*) [*phi^+^*] *jjj3*::*LEU2*	Sahi and Craig (2007)
*jjj1*Δ	*MATa/*α *his3*Δ*1/his3*Δ*1 leu2*Δ*0/leu2*Δ*0 LYS2/lys2*Δ*0 met15*Δ*0/MET15 ura3*Δ*0/ura3*Δ*0 jjj1*::*KanMX4*	Thermo yeast knockout collection
*swa2*Δ	*MATa/*α *his3*Δ*1/his3*Δ*1 leu2*Δ*0/leu2*Δ*0 LYS2/lys2*Δ*0 met15*Δ*0/MET15 ura3*Δ*0/ura3*Δ*0 swa2*::*KanMX4*	Thermo yeast knockout collection
*cwc23*Δ	*MAT*α *leu2-3*,*112 trp1-1 can1-100 ura3-1 ade2-1 his3-11*,*15* [*phi^+^*] cwc23::*KanMX4/URA-pRS316-Cwc23*	Sahi *et al.* (2010)
*sis1*Δ	[*RNQ^+^*] [*psi^−^*] [*p316-SIS1-Sis1*] *sis1*::*LEU2 ade2-1 ura3-52 leu2-3*, *112 trp1-289 his3-200*	Lopez *et al.* (2003)
*sis1*Δ*ydj1*Δ	*MAT*α*leu2-3*,*112 trp1-1 can1-100 ura3-1 ade2-1 his3-11*,*15* [*phi^+^*] *ydj1*::*LEU2 sis1*::*LEU2/pYW17* (SIS1*-YCp50*)	Johnson and Craig (2001)
*xdj1*Δ*pam17*Δ	*MAT*α*leu2-3*,*112 trp1-1 can1-100 ura3-1 ade2-1 his3-11*,*15* [*phi^+^*] x*dj1*::*LEU2 pam17*::*HYG*	Sahi *et al.* (2013)
*apj1*Δ*slx5*Δ	*MATa*(*leu2-3*,*112 trp1-1 can1-100 ura3-1 ade2-1 his3-11*,*15*) [*phi^+^*] *apj1*::*HIS3 slx5*::*KanMX4*	Sahi *et al.* (2013)
[*PSI^+^*]	[*PSI^+^*] [*rnq^−^*] [*p316-SIS1-Sis1*] *sis1*::*LEU2 ade1-14 ura3-52 leu2-3*, *112 trp1-289 his3-200*	Harris *et al.* (2014)
[*URE3*]	[*URE3-1*], [*p316-SWA2-Swa2*], *swa2*::*HIS3*, *trp1-1*, *ura3-1*, *leu2-3*,*112*, *ade2-1*, *his3-11*,*15*, *dal5*::*PDAL5-ADE2*	Troisi *et al.* (2015)

### Western analysis

Total proteins were isolated from an equal number of cells growing in exponential phase by treating cells with 0.1 M NaOH and resuspended in SDS sample buffer (62.5 mM Tris·HCl, pH 6.8, 5% glycerol, 2% SDS, 2% β-mercaptoethanol, and 0.01% bromophenol blue). Protein was detected by using anti-Mdj1 (a gift from Elizabeth Craig, UW-Madison, WI) or anti-HA (Sigma-Aldrich) rabbit antibodies. Quantification was done with ImageQuant software (Molecular Dynamics, Sunnyvale, CA). Semidenaturing detergent agarose gel electrophoresis (SDDAGE) was used to confirm the presence of both [*RNQ*^+^] and [*PSI*^+^] (Aron *et al.* 2007; Harris *et al.* 2014). Briefly, cells were lysed by vortexing at 4° with sterile glass beads. Following centrifugation at 4°, cleared lysates were mixed with SDS loading buffer and incubated at 25° for 7–8 min. Aggregates were resolved in a 1.5% (w/v) Tris-glycine (0.1% SDS) agarose gel (SeaKem Gold PFGE agarose) and protein was transferred to a nitrocellulose membrane at 1A for 1 hr at 25° in a Tris-glycine/methanol buffer. Prion aggregates were visualized by performing western analysis using antibodies specific for either Rnq1 or Sup35 (gifts from the Craig and Tuite labs, respectively).

### Plant methods

Wild-type *A. thaliana* (Col-0) plants were grown on full-strength MS-agar medium supplemented with 0.5% sucrose and 0.05% MES [(2-(N-morpholino) ethanesulfonic acid] at 20° under a 16 hr d (140 µmol m^−2^ sec^−1^) and 8 hr night regime. After germination, 20–25 d later, plantlets were subjected to various stress treatments. Mechanical stress was inflicted by puncturing leaves by three to five consecutive applications of 10 pins together and, subsequently, the plantlets were left on the media plates for 1 hr. Different developmental tissues were harvested at various growth stages. Tissues were frozen in liquid nitrogen and stored at −80° until use. Next, 1 µg of total RNA isolated (Spectrum plant total RNA kit, Sigma-Aldrich) was reverse transcribed using an iScript cDNA synthesis kit (BioRad), according to the manufacturer’s instructions. Primers were designed using a very stringent set of criteria (Czechowski *et al.* 2004). Real-time PCR was performed in a CFX connect 96-well real-time system using iTaq universal SyBr green Supermix (BioRad). Data analysis was done according to MIQE guidelines (Bustin *et al.* 2009).

### Data availability

Plasmid constructs and yeast strains are available upon request. The authors state that all data necessary for confirming the conclusions presented in the article are represented fully within the article.

## Results

### Functional specificity of J proteins is conserved

**Figure 1:**
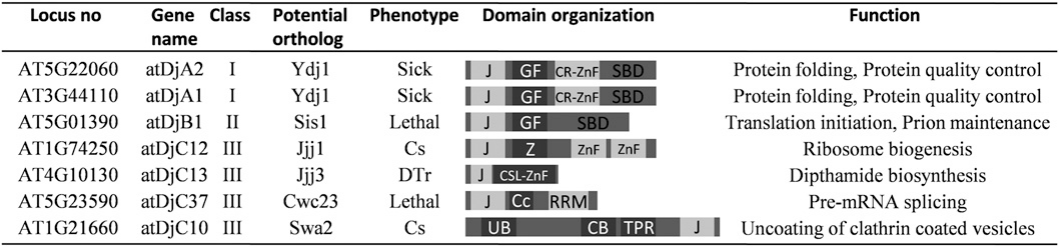
J protein orthologs in *A. thaliana* used in this study. Domain organization of cytosolic J proteins in *A. thaliana* that were predicted to be orthologs of J proteins of *S. cerevisiae*. CB, clathrin binding; Cc, coiled coil region; CR-ZnF, cysteine-rich zinc finger; Cs, cold sensitive; CSL-Znf, CSL-type zinc finger; DTr, dipthamide toxin resistance; GF, glycine phenylalanine-rich region; J, J domain; RRM, RNA recognition motif; SBD, substrate-binding domain; Sick, slow growing; TPR, tetratricopeptide repeat; UB, ubiquitin association; Z, Zuotin-like region; ZnF, zinc finger.

To understand the conservation and diversity of the J protein network in *A. thaliana*, we took advantage of our detailed understanding of the J protein network in the unicellular eukaryote *S. cerevisiae*. The cytosol of *S. cerevisiae* is home to the most complex and well-understood Hsp70:J protein network. We concentrated our study to J proteins Ydj1, Sis1, Jjj1, Jjj3, Cwc23, and Swa2, because all these work with the same multi-functional Hsp70, Ssa, to perform diverse cellular functions (Figure 1). Further, their deletions result in assayable phenotypes in *S. cerevisiae*, making functional analysis possible (Sahi and Craig 2007). Using the PSI-BLAST approach, we identified at least one cytosolic ortholog of each of the J proteins—Ydj1, Sis1, Jjj1, Jjj3, Cwc23, and Swa2—in the *Arabidopsis* cytosol (Figure 1). The identified plant orthologs contained all the characteristic domains and motifs present in their corresponding yeast counterparts (Figure 1). Over long evolutionary timescales, orthologous proteins retaining similar sequences usually continue to perform the same protein function that was originally present in the ancestral protein (Lee *et al.* 2007). However, under selective constrains, they may accumulate mutations resulting in functional diversity that is useful to the organism (Torgerson and Singh 2004). Consequently, we asked if these orthologs carry the same chaperone functions as their yeast counterparts. To test their functionality, full-length ORFs of *Arabidopsis* J proteins atDjA1, atDjA2, atDjB1, atDjC12, atDjC13, atDjC10, and atDjC37 were cloned in yeast expression vectors driven by a promoter of moderate strength, *TEF* (Table 1). Yeast strains carrying the respective J protein deletions (Table 2) were transformed with all these constructs and analyzed for J protein functions.

**Figure 2:**
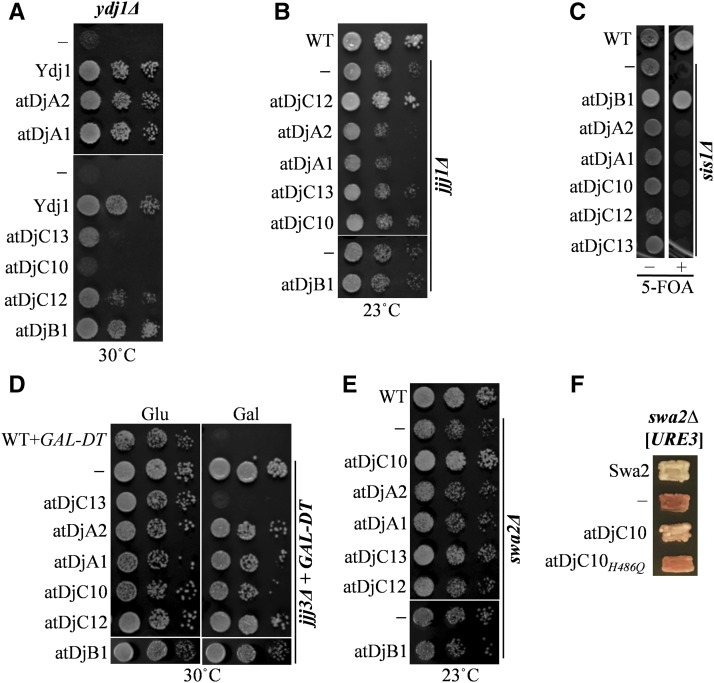
Functional specificity of J protein orthologs of *A. thaliana*. (A, B, and E) Five microliters of 10-fold serial dilutions of wild-type cells harboring empty pRS413 plasmid (WT) or J protein deletion strains, *ydj1*Δ, *jjj1*Δ, or *swa2*Δ, transformed with either empty plasmid (−) or various J protein-expressing constructs were spotted on histidine (His) drop-out plates and incubated at indicated temperatures. (C) Five microliters of WT cells harboring empty pRS413 plasmid (WT) or *sis1*Δ cells transformed with either empty plasmid or different J protein-expressing constructs was spotted on media with (+) or without (−) 5-fluoroorotic acid (5-FOA) and incubated at 30° for 3 d. (D) Five microliters of 10-fold serial dilutions of WT cells harboring *GAL-DT* along with an empty pRS413 plasmid (WT-*GAL-DT*) and *jjj3*Δ cells either with an empty plasmid (−) or different J protein expressing constructs were spotted on plates containing glucose (Glu) and galactose (Gal) and incubated at 30° for 3 d. (F) [*URE3*] *swa2*∆ cells expressing Swa2 from a *URA3*-marked plasmid (pRS-315-*SWA2*-Swa2) were transformed by plasmids expressing Swa2, atDjC10, atDjC10*_H486Q_*, or empty plasmid (−), subjected to plasmid shuffling on 5-FOA, and finally assayed for the continued maintenance of the prion following loss of the *URA3*-marked plasmid. Cells expressing full-length Swa2 are used as a positive control for the stability of the prion throughout the plasmid-shuffling and prion-detection procedures. The maintenance of [*URE3*] was assayed by colony color on rich medium and are shown for representative transformants (*n* = 16): in this genetic background, [*URE3*] cells form white colonies whereas [*ure-o*] cells lacking the prion form red colonies.

Ydj1 is the major cytosolic J protein in budding yeast and is important for growth at elevated temperatures, as *ydj1*Δ strains are slow growing and exhibit a “sick” phenotype (Sahi and Craig 2007). When expressed from a *TEF* promoter, both atDjA1 and atDjA2 rescued the sick phenotype of *ydj1*Δ like full-length Ydj1 (Figure 2A and Figure S1 in File S1). Next, we began to investigate the functionality of other more specialized J protein orthologs. Jjj1 is a class III J protein that associates with the ribosome and plays an important and unique role in the biogenesis of the 60S ribosomal subunit. Yeast cells lacking Jjj1 show cold sensitivity at 23° and aberrant polysome profiles (Demoinet *et al.* 2007; Meyer *et al.* 2007). AtDjC12, the predicted ortholog of Jjj1 in *A. thaliana*, when expressed in the *jjj1*Δ strain, completely rescued the cold sensitivity of *jjj1*Δ cells (Figure 2B). Similarly, atDjB1 was identified as an ortholog of Sis1 in *A. thaliana*. Sis1 is an essential, cytosolic class II J protein of *S. cerevisiae*. It is involved in translation initiation, remodeling of protein aggregates, and the degradation of misfolded proteins (Arndt *et al.* 1989; Park *et al.* 2013; Summers *et al.* 2013). We tested if atDjB1 could substitute for Sis1 by using the 5-FOA plasmid shuffling method. *sis1*Δ cells expressing atDjB1 from a *CEN-TEF* plasmid could lose the *URA3*-marked Sis1 plasmid on 5-FOA, suggesting that atDjB1 carries same essential function as Sis1 (Figure 2C). Jjj3 is a specialized class III J protein essential for diphthamide (DPH) biosynthesis in yeast (Chen *et al.* 1985). DPH is a unique, post-translationally modified histidine residue present in eukaryotic elongation factor 2 (Schaffrath *et al.* 2014). Paradoxically, this same modification makes the eEf2 protein sensitive to diphtheria toxin (DT), in which ADP ribosylates the eEf2 molecule at the DPH residue rendering it nonfunctional, thus stopping protein synthesis (Schaffrath *et al.* 2014). In the presence of the *GAL-DT* plasmid and on galactose-containing medium, *jjj3*Δ cells having a functional copy of Jjj3 are dead. *jjj3*Δ [*GAL-DT*] cells expressing atDjC13, the ortholog of Jjj3, were dead on galactose-containing medium like wild-type cells, demonstrating that atDjC13 can restore DPH biosynthesis in *jjj3*Δ cells (Figure 2D). Swa2 is the yeast homolog of the human J protein, auxilin, that along with cytosolic Hsp70, functions in the uncoating of clathrin-coated vesicles, has an important role in endocytosis (Pishvaee *et al.* 2000), and has been identified as a critical factor for the maintenance of the prion [*URE3*] (Troisi *et al.* 2015). *S. cerevisiae* cells lacking Swa2 are slow growing, sensitive to low temperatures, and fail to propagate [*URE3*] (Troisi *et al.* 2015; Xiao *et al.* 2006). atDjC10 was identified as the *A. thaliana* ortholog of Swa2. *swa2*Δ cells harboring atDjC10 grew comparable to the wild-type cells at 23°, indicating that atDjC10 was able to fully substitute for yeast Swa2 in restoring normal growth rate (Figure 2E). Similarly, atDjC10 was able to substitute for yeast Swa2 in the maintenance of [*URE3*], albeit with reduced stability of the prion (Figure 2F). Given the dramatic sensitivity of [*URE3*] to even very small alterations in chaperone protein function (Higurashi *et al.* 2008; Hines and Craig 2011; Reidy *et al.* 2014; Sporn and Hines 2015; Troisi *et al.* 2015), this result indicates a very high level of functional complementation by the plant homolog. Cwc23 is an essential J protein of *S. cerevisiae* that interacts with spliceosomal proteins and is important for pre-mRNA splicing (Pandit *et al.* 2009; Sahi *et al.* 2010). atDjC37 was identified as an ortholog of Cwc23 in *A. thaliana*. The viability of the yeast Cwc23 deletion strain is maintained by a wild-type copy of Cwc23 expressed from a *URA3*-marked plasmid. *cwc23*Δ cells expressing atDjC37 from a *CEN-TEF* plasmid were unable to lose the *URA3*-marked Cwc23 plasmid on 5-FOA medium, suggesting that atDjC37 could not replace Cwc23 in *S. cerevisiae* (data not shown). Although it is premature to speculate the possible reasons behind this, we hypothesize that atDjC37 has diverged significantly as compared to Cwc23 of *S. cerevisiae* and, thus, is unable to work in the yeast system. HA-tagging of these constructs followed by western analysis revealed that all the *A. thaliana* J proteins were expressed at similar levels in yeast cells (Figure S2 in File S1). Therefore, our results show that all potential J protein orthologs from *Arabidopsis*, except that of Cwc23, could functionally substitute their corresponding orthologs in yeast.

**Figure 3:**
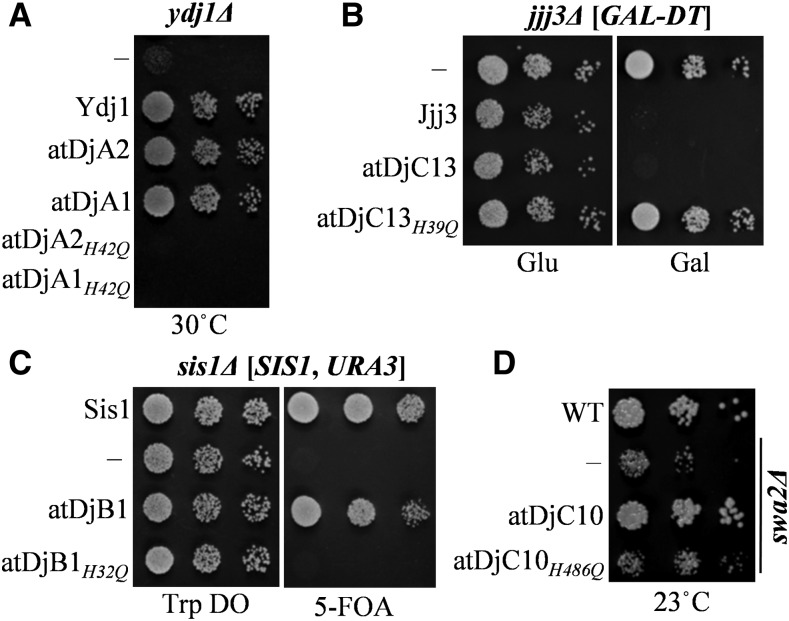
Functionality of *A. thaliana* J proteins requires J domain. (A) Five microliters of 10-fold serial dilutions of *ydj1*Δ transformed with either empty pRS413 plasmid (−), pRS413-*TEF*-Ydj1 (Ydj1), or J protein-expressing plasmids [pRS413-*TEF*-atDjA2 (atDjA2), pRS413-*TEF*-atDjA1 (atDjA1), pRS413-*TEF*- pRS413-*TEF*-atDjA2*_H42Q_* (atDjA2*_H42Q_*), or atDjA1*_H42Q_* (atDjA1*_H42Q_*)] were spotted on His (histidine) drop-out plates and incubated at 30° for 3 d. (B) Five microliters of 10-fold serial dilutions of *jjj3*Δ cells either with an empty pRS413 plasmid (−) or J protein-expressing plasmids [pRS413-*TEF*-Jjj3 (Jjj3), pRS413-*TEF*-atDjC13 (atDjC13), and pRS413-*TEF*-atDjC13*_H39Q_* (atDjC13*_H39Q_*)] were spotted on His drop-out plates containing either glucose (Glu) or galactose (Gal) and incubated at 30° for 3 d. (C) Five microliters of *sis1*Δ cells transformed with empty pRS414 plasmid (−) or J protein-expressing plasmids [pRS414-*TEF*-Sis1 (Sis1), pRS414-*TEF*-atDjB1 (atDjB1), and pRS414-*TEF*-atDjB1*_H32Q_* (atDjB1*_H32Q_*)] were spotted on tryptophan (Trp) drop-out plates with (+) or without (−) 5-fluoroorotic acid (5-FOA) and incubated at 30° for 3 d. (D) Five microliters of 10-fold serial dilutions of wild-type cells harboring pRS413 empty plasmid (WT) and the *swa2*Δ strain either harboring an empty pRS413 plasmid (−) or J protein-expressing constructs [pRS413-*TEF*-atDjC10 (atDjC10) or pRS413-*TEF*-atDjC10*_H486Q_* (atDjC10*_H486Q_*)] were spotted on His drop-out plates and incubated at 23° for 3 d.

All the J proteins analyzed, except Ydj1, are highly specialized in their functions (Sahi and Craig 2007). Therefore, we next asked if the plant J protein orthologs also carry the same specificities as their yeast counterparts. To access this, the J protein-expressing plasmids were now transformed into all other yeast J protein deletion strains. As expected, none of yeast deletion strains, except Ydj1, were rescued by *Arabidopsis* genes other than their own orthologs (Figure 2, A–E) indicating high conservation in their functional specificity. Ydj1 is a generalized J protein involved in many cellular processes. Overexpression of other J proteins, including Sis1, is known to rescue the slow growth of *ydj1*Δ (Sahi and Craig 2007; Yan and Craig 1999). Rescue of temperature sensitivity of *ydj1*Δ by atDjB1, a Sis1 ortholog in *Arabidopsis*, further validates that such functional redundancies possibly exist in atDjB1 as well. Further, we show that the rescue of the respective J protein deletion strains required a functional J domain, as an H to Q mutation in the HPD tripeptide motif in the respective J domains, which is critical for interaction with Hsp70, completely abolished the function of the plant J protein orthologs tested (Figure 3, A–D). Likewise, mutation of the HPD motif in atDjC10 (the homolog of yeast Swa2) abolished its ability to maintain the prion [*URE3*] (Figure 2F). These results suggest that the ability of plant J protein orthologs to rescue the corresponding J protein deletion phenotypes was dependent on their interaction with the yeast Hsp70. Put together, our data shows that the functional specificities of the overall cytosolic J protein complement involved in fundamental chaperone functions is retained between *S. cerevisiae* and *A. thaliana*. This clearly suggests that Hsp70:J protein machines involved in fundamental chaperone functions, such as cytosolic protein quality control, 60S ribosome biogenesis, translation initiation, DPH biosynthesis, and clathrin mediated endocytosis are evolutionarily conserved.

### Expansion of the J protein network in plants

Yeast complementation data showed that most of the orthologous J proteins in *A. thaliana* with highest sequence similarity to their yeast counterparts also displayed functional similarity. However, because the of significant increase in the number of J proteins in plants, it would be inappropriate to conclude that the whole J protein chaperone complement in *Arabidopsis* is like the one in *S. cerevisiae*. To get a better idea of the existing J protein network in plants, we performed a detailed study of all J proteins present in *A. thaliana*. The *A. thaliana* genome was examined for loci encoding J domain-containing proteins. Using the best BLAST hit (BBH) approach (*see* Method S1 in File S1), a total of 106 J proteins were identified (Table S2 in File S1), out of which 71 were predicted to be nucleo–cytoplasmic while 35 of them showed greater propensity to be localized to the endoplasmic reticulum, plastid, or mitochondria (Table S2 in File S1). This is by far the most updated and accurate list of J proteins encoded by the *Arabidopsis* genome. The Hsp70 number in the *A. thaliana* cytosol is seven, as compared to six in the single-celled eukaryote *S. cerevisiae* (Boorstein *et al.* 1994; Sung *et al.* 2001). A more significant increase in the number of J protein-encoding genes as compared to Hsp70s is consistent with the idea that the functional diversity of Hsp70:J protein machines predominantly comes from J proteins. With respect to *S. cerevisiae*, an interesting distribution of J protein complement was observed in the *Arabidopsis* genome. While some J proteins such as Cwc23, Jjj1, and Jjj3 maintained their singularity in the *A. thaliana* genome, others seem to be present in multiple numbers *viz*. Sis1, Swa2, and Ydj1. Additionally, orthologs of Xdj1 and Apj1 could not be identified. A total of 43 cytosolic J proteins could not be placed in any orthologous context and appeared to be novel innovations in the *Arabidopsis* genome. Taken together, our *in silico* analysis revealed a very complex scenario of J proteins in the *A. thaliana* cytosol, suggesting that the J protein network in the *Arabidopsis* cytosol is expanding, which might translate into increased complexity of chaperone functions in higher plants.

**Figure 4:**
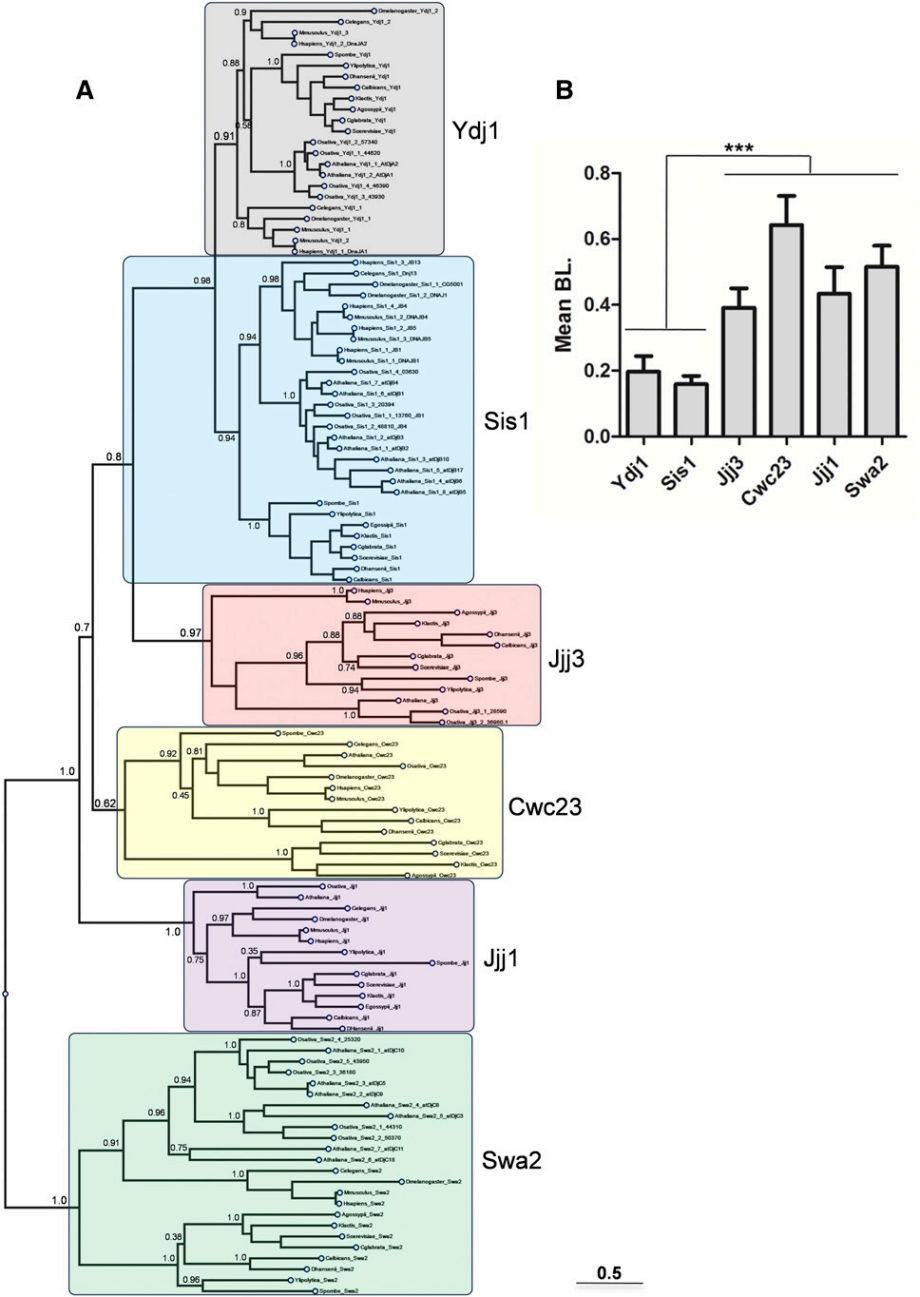
Phylogenetic distribution of cytosolic J proteins orthologs. (A) Phylogenetic tree of six J protein orthologs from plants, animals, and fungi. Unrooted approximately-maximum-likelihood tree generated in FastTree with 70% or above SH-like support values (Shimodaira-Hasegawa-like procedure) indicated for each clade. Number of sequences sampled for each gene is mentioned inside the triangle. The scale bar represents estimated substitutions per site. (B) Comparative evolutionary rate of J protein orthologs. Terminal branch length values obtained from phylogenetic tree were used to evaluate mean branch length (BL) of different J protein orthologous group and depicted as bar graph. Values are measured as mean ± SD. Merged values of class III J proteins are significantly different from Ydj1 and Sis1at the *P* < 0.0001 level (Table S4 in File S1).

To further address the emerging complexity of the cytosolic J protein network in *A. thaliana*, we performed a comprehensive and rigorous phylogenetic analysis. We again concentrated our analysis to only Ydj1, Sis1, Jjj1, Jjj3, Cwc23, and Swa2. For this, a total of 118 protein sequences (orthologs of Ydj1, Sis1, Jjj1, Jjj3, Cwc23, and Swa2) from 14 taxa, covering three major groups of organisms—including eight fungal, four animals, and two plants species—were analyzed (Table S3 in File S1). The orthologous relationship of all identified J proteins was established by their close phylogenetic relationship with the respective *S. cerevisiae* J proteins, as well as other J proteins of the same family (Figure 4A). All of the analyzed *Arabidopsis* proteins clustered into distinct clades forming individual orthologous groups resulting in a topology resembling a polyphyletic gene-tree (Figure 4A). Further, the taxonomic hierarchy was maintained within each clade, suggesting that a group of monophyletic J protein sequences belonging to a distinct class of J protein have descended from the ancestral sequence at the root of each clade. Unlike other J proteins, where the J domain is at the N-terminus, the J domain in Swa2 is positioned at the C-terminus, thus Swa2 orthologs formed a discrete clade that appeared as an out-group.

Following phylogenetic tree construction, evaluation of branch lengths can give an estimate of evolutionary rates of proteins, which is instrumental in understanding the conservation as well as the diversity of biochemical processes. To get an idea about the diversity of J protein functions in the cytosol, we calculated the evolutionary rates of Ydj1, Sis1, Jjj1, Jjj3, Cwc23, and Swa2 using average branch lengths within each orthologous group. Our results show that, as compared to Ydj1 and Sis1, orthologs of Jjj1, Jjj3, Cwc23, and Swa2 are evolving much faster (Figure 4B). Among all the J proteins analyzed, Cwc23 was the fastest evolving (Table S4 in File S1). Orthologs usually result from the divergence of homologous genes through speciation, while divergence of orthologs through gene duplication followed by speciation leads to the formation of paralogs (Lee *et al.* 2007). Our phylogenetic data show that Sis1 and Swa2 have duplicated in *Arabidopsis* after the species split and thus are in-paralogs. In contrast, atDjA1 and atDjA2 appear to be out-paralogs that arose by gene duplication before speciation. Orthologs of Jjj1 and Jjj3 continue to stay as singletons in *Arabidopsis* through speciation. Apart from this, Cwc23, which is essential in *S. cerevisiae*, also remained as a single-copy gene in *Arabidopsis*, unlike most other essential genes. It seems that Cwc23 manages to evolve without gene duplication by accumulating beneficial mutations in its sequence, which is reflected by its fast rate of evolution. Although it is likely that paralogs have redundant functions, over time they may exhibit a shift in their ancestral functions by retaining a subset of their original functions leading to subfunctionalization. Additionally, paralogs are also known to acquire completely new biochemical activities resulting in neofunctionalization.

### Gene duplication resulting in functional diversification

**Figure 5:**
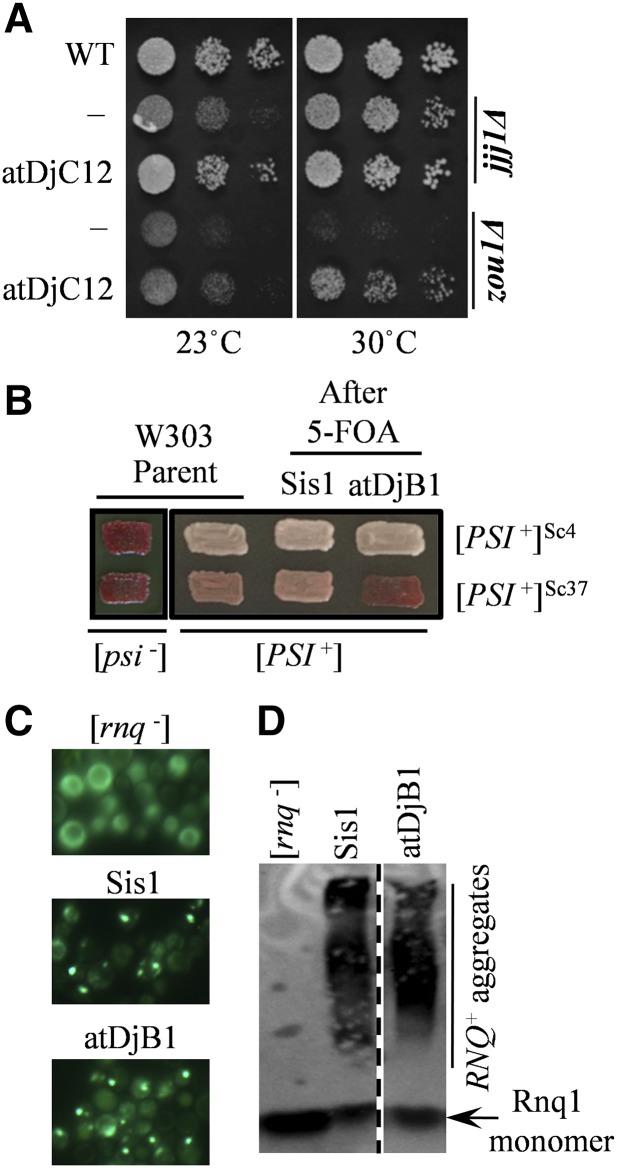
Functional diversity in *A. thaliana* J proteins. (A) Five microliters of 10-fold serial dilution of wild-type cells harboring an empty pRS413 plasmid (WT), or *jjj1*Δ and *zuo1*Δ cells transformed either with empty pRS413 plasmid (−) or pRS413-*TEF*-atDjC12 (atDjC12), were spotted on His (histidine) drop-out plates and incubated at indicated temperature for 3 d. (B–D) [*PSI*^+^]^Sc4^, [*PSI*^+^]^Sc37^, or [*RNQ*^+^] cells were transformed by plasmids expressing atDjB1, pRS414-*TEF*-atDjB1 (atDjB1) or Sis1, pRS313-*SIS1*-Sis1 (Sis1), subjected to plasmid shuffling on 5-fluoroorotic acid (5-FOA), and finally assayed for the continued maintenance of the prion following loss of the *URA3*-marked plasmid (pRS316-*SIS1*-Sis1). Cells expressing full-length Sis1 are used as a positive control for the stability of the prion throughout the plasmid-shuffling and prion-detection procedures. GdnHCl-treated [*psi^−^*] parent strains are included for comparison. (B) The maintenance of [*PSI*^+^] was assayed by colony color on rich medium. Color phenotype assays are shown for representative transformants (*n* ≥ 10) with parental strains included for comparison. (C) The maintenance of [*RNQ*^+^] was assayed by subsequent transformation of each shuffled strain by an Rnq1-GFP (green fluorescent protein) reporter plasmid (pRS416-*CUP1*-Rnq1-GFP) followed by fluorescence microscopy analysis. Fluorescence patterns indicative of [*RNQ*^+^] maintenance (punctate) or loss (diffuse) are shown for representative transformants (*n* ≥ 10) with control [*rnq*^−^] cells included for comparison. (D) The maintenance of [*RNQ*^+^] was further confirmed by semidenaturing detergent agarose gel electrophoresis (SDDAGE). Detergent-resistant Rnq1 aggregates indicative of the presence of [*RNQ*^+^] were resolved by SDDAGE and visualized by immunoblot analysis using antibodies specific for Rnq1. Control [*rnq*^−^] cells were included for comparison.

Gene duplication relaxes evolutionary constraints often leading to functional diversity in orthologous proteins (Koonin 2005). While some J proteins were found to have proliferated, such as Sis1, Swa2, and Ydj1, only one BBH was identified for proteins like Jjj1, Jjj3, and Cwc23. After establishing *A. thaliana* cytosolic J proteins in their orthologous context, we asked whether gene proliferation translated into any divergence in protein function. We chose two extreme cases, Sis1 and Jjj1. The *A. thaliana* genome encodes eight potential orthologs of Sis1 and a single Jjj1. Interestingly, both Sis1 and Jjj1 are known to carry out more than one function in the yeast cytosol. Besides its role in biogenesis of the 60S ribosomal subunit, overexpression of Jjj1 also rescues the slow growth phenotype of cells lacking Zuo1, another ribosome-associated J protein (Meyer *et al.* 2007). Zuo1 works with the specialized Hsp70, Ssb1/2, and is involved in nascent protein folding and ribosome biogenesis (Albanese *et al.* 2010). We asked if atDjC12, which rescued the growth defect of a *jjj1*Δ strain, could also rescue *zuo1*Δ. When overexpressed from a *TEF* promoter, atDjC12 rescued the growth defect of a *zuo1*Δ strain, as compared to vector-transformed cells (Figure 5A), suggesting that atDjC12 might be involved in nascent polypeptide folding in addition to 60S ribosomal subunit biogenesis in *A. thaliana*.

Sis1, in addition to its essential function in *S. cerevisiae*, is also involved in the remodelling of protein aggregates, which makes it indispensable for the propagation of all major yeast prions (Arndt *et al.* 1989; Aron *et al.* 2007; Higurashi *et al.* 2008; Hines *et al.* 2011; Sondheimer *et al.* 2001). Therefore, we asked whether the biochemical activity of Sis1, which enables it to remodel prion aggregates, is also evolutionarily conserved in the *A. thaliana* protein atDjB1. Prions formed from the same protein can exist as distinct structural conformers (amyloid polymorphisms), which are known as prion “strains”, that determine species barriers and pathological progression or “variants” in yeast, which affect mitotic stability, chaperone interactions, and the extent or “strength” of the associated phenotypes (Liebman and Chernoff 2012). As such, prion variants in yeast are typically classified as “strong” or “weak” on the basis of phenotypic strength and mitotic stability. We examined the potential for atDjB1 to propagate two distinct and well-studied variants of the prion [*PSI*^+^]: [*PSI*^+^]^Sc4^, a strong variant, and [*PSI*^+^]^Sc37^, a weak variant (Tanaka *et al.* 2006). To determine whether atDjB1 can substitute for Sis1 in prion propagation, we first transformed [*PSI*^+^] *sis1*Δ cells expressing Sis1 from a *URA3*-marked plasmid with a *CEN-TEF* plasmid expressing atDjB1, or another plasmid expressing Sis1 as a positive control, and again plated cells on 5-FOA to allow for loss of the *URA3*-marked Sis1 plasmid. In these strains, [*PSI*^+^] cells can be distinguished from cells lacking the prion ([*psi*^−^] cells) by a well-established colony color assay; on rich, glucose-based, solid media [*PSI*^+^] cells form light pink (strong variants) or dark pink (weak variants) colonies, whereas [*psi*^−^] cells form red colonies. atDjB1 was able to replace Sis1 in the propagation of strong, but not weak, [*PSI*^+^] variants (Figure 5B). We also determined whether atDjB1 was able to propagate a strong variant of [*RNQ*^+^] called [*RNQ*^+^]^STR^ (Harris *et al.* 2014). To do this, following the replacement of Sis1 with atDjB1 in [*RNQ*^+^]^STR^ cells, strains were transformed again with a plasmid expressing the prion-forming protein Rnq1 tagged with green fluorescent protein (Rnq1-GFP) and the continued presence of [*RNQ*^+^] was determined by fluorescence microscopy: [*RNQ^+^*] cells expressing Rnq1-GFP exhibit heterogeneous (punctate) fluorescence patterns as the fluorescent chimera is recruited into preexisting prion aggregates while in [*rnq*^−^] cells the fluorescence is homogenously distributed about the cytoplasm (diffuse fluorescence) (Figure 5C). atDjB1 maintained [*RNQ*^+^], similar to Sis1 (Figure 5C). To confirm that our fluorescence assay accurately reports the maintenance of the prion, rather than the presence of nonamyloid amorphous aggregates, we conducted a biochemical assay, SDDAGE, which resolves detergent-resistant amyloid aggregates on the basis of size (Kryndushkin *et al.* 2003). Lysates of representative transformants were subjected to SDDAGE immunoblot analysis with an antibody specific for Rnq1 protein; as expected, in control [*rnq*^−^] cells, Rnq1 exists only as a monomer and migrates far into the gel, whereas in presumed [*RNQ*^+^] cells expressing either Sis1 or atDjB1, Rnq1 additionally exists in high molecular weight, detergent-resistant aggregates, consistent with the maintenance of the prion in these strains (Figure 5D). Taken together, these results demonstrate that atDjB1 has maintained the same prion-propagating functions of Sis1 as previously determined for both the human and fruit-fly homologs (Harris *et al.* 2014; Lopez *et al.* 2003). These results suggest that, while the essential functions of Sis1 are also maintained in atDjB1, its ability to propagate different prion variants is not equally maintained by the *Arabidopsis* ortholog. Put together, our results thus show that, while atDjC12 was able to carry out all functions of Jjj1, duplication of Sis1 orthologs in *A. thaliana* might have led to the functional diversification observed in atDjB1.

### Gene sharing and regulatory differences in J protein orthologs

The results presented above suggested that plant J proteins have functionally diverged resulting in a greater diversity of Hsp70:J protein machines in plants. Emergence of functional diversification in proteins depends on a lot of factors, including but not limited to gene duplication, expression levels, and the number of interacting partners (Gabaldon and Koonin 2013). We set out to investigate the emergence of evolutionary novelties in plant J proteins more rigorously. We chose to study atDjA1 and atDjA2, the two identified Ydj1 orthologs in *A. thaliana*, because Ydj1 has been functionally and evolutionarily characterized in significant detail. Phylogenetic analysis of Ydj1 showed that, unlike other cytosolic J proteins that formed species clades, the distribution of Ydj1 homologs formed genic clades. Ydj1 homologs appeared to have duplicated first, followed by speciation. This suggested that atDjA1 and atDjA2 might have different functions in *Arabidopsis*.

**Figure 6:**
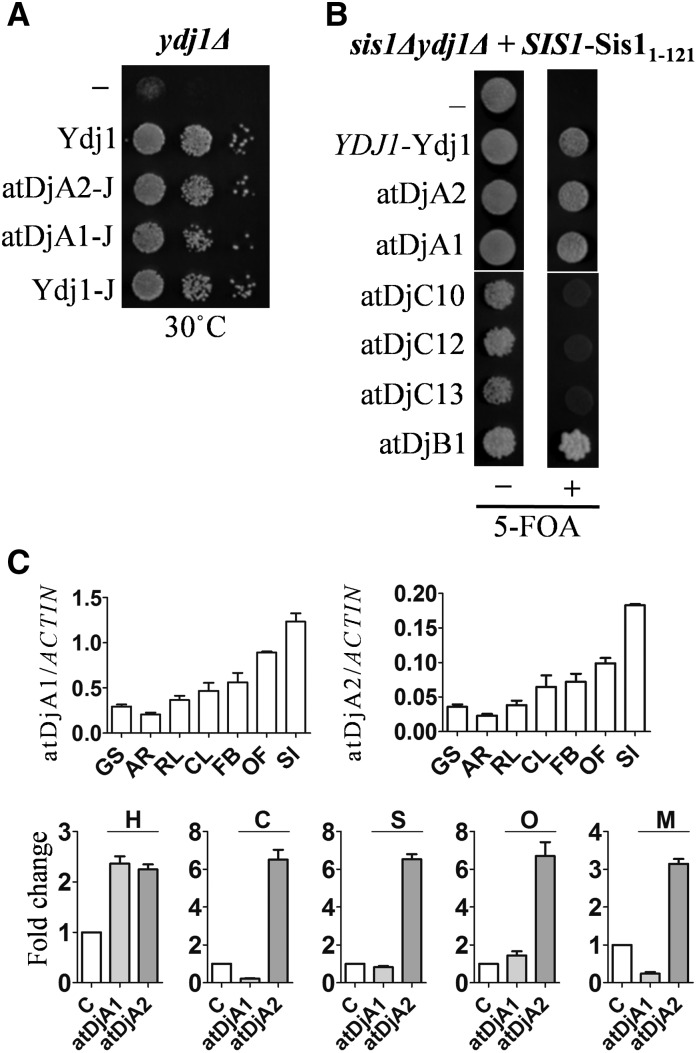
atDjA2 and atDjA1 are orthologs of Ydj1. (A) Five microliters from 10-fold serial dilutions of *ydj1*Δ cells harboring empty pRS413-*TEF* vector (−), pRS413-*TEF*-Ydj1 (Ydj1), or J domain-containing fragments of atDjA2_1–128_ (atDjA2-J), atDjA1_1–127_ (atDjA1-J), or Ydj1_1–220_ (Ydj1-J) expressed from a pRS413-*TEF* plasmid were spotted on His (histidine) drop-out plates and incubated at the indicated temperature for 3 d. (B) Five microliters of *sis1*Δ*ydj1*Δ [*SIS1*, *URA3*] pRS313-Sis1_1–121_ cells transformed with either an empty pRS414-*TEF* plasmid (−) or different J protein-expression plasmids, pRS314-Ydj1 (*YDJ1*-Ydj1), pRS414-*TEF*-atDjA2 (atDjA2), pRS414-*TEF*-atDjA1 (atDjA1) pRS414-*TEF*-atDjC10 (atDjC10), pRS414-*TEF*-atDjC12 (atDjC12), pRS414-*TEF*-atDjC13 (atDjC13), and pRS414-*TEF*-atDjB1, were spotted on tryptophan (Trp) drop-out plates with (+) or without 5-fluoroorotic acid (5-FOA) (−) and incubated at 30° for 3 d. (C) cDNA prepared from total RNA isolated from different tissues, upper panel: germinating seeds (GS), adult roots (AR), rosetta leaves (RL), cauline leaf (CL), flower buds (FB), open flowers (OF), and siliques (SL). Stressed samples, lower panel: control untreated *Arabidopsis* seedlings (open bars) or seedlings treated (gray bars) with: 37° for 60 min (H); 4° for 24 hr (C); 100 mM sodium chloride, 24 hr (S); 150 mM Mannitol, 12 hr (O); or mechanical injury, 60 min (M). Samples were subjected to real-time PCR using primers specific for the J proteins indicated. Gene expression levels in stressed samples were normalized to the expression levels of respective genes in unstressed samples. All results were normalized against the expression level of *ACTIN* (At3G18780) gene. Data are mean ± SD of two biological and technical replicates.

Both atDjA1 and atDjA2 rescued the temperature sensitivity of *ydj1*Δ in a J domain-dependent manner (Figure 3A). However, this experiment does not firmly establish that atDjA1 and atDjA2 have similar functions to Ydj1, as the growth defects of *ydj1*Δ can also be significantly rescued by various other J proteins and J domain-containing fragments (Sahi and Craig 2007). As such, we first tested the ability of J domain fragments of atDjA1 and atDjA2 to rescue *ydj1*Δ. When expressed from a moderate-strength *TEF* promoter, both atDjA1-J and atDjA2-J rescued the temperature sensitivity of *ydj1*Δ at 30° (Figure 6A). This was comparable to the rescue obtained by full-length or the J domain fragment of Ydj1 (Figure 6A). The nonspecificity of the sick phenotype of *ydj1*Δ was further supported by our result that when mis-localized to the cytosol, the J domain-containing fragment of mitochondrial J protein, Mdj1, which does not normally work with the cytosolic Hsp70 Ssa, also rescued the growth defects of *ydj1*Δ (Figure S3 in File S1).

To establish whether atDjA1 and atDjA2 are Ydj1-like proteins and that the ability of these two proteins to replace Ydj1 was not just due to their ability to stimulate the ATPase activity of Ssa, another strategy was used. Sis1 is an essential J protein in yeast, and a Sis1_1–121_ fragment, containing the J domain and the following GF/GM region of Sis1, is sufficient for cell viability, but only in the presence of full-length Ydj1; Ydj1 and Sis1 have some overlapping functions, as the C-terminal CBD of either Ydj1 or Sis1 is required for viability. This phenotype is specific for Ydj1 as the J domain fragment is not sufficient to rescue the viability of a *sis1*Δ*ydj1*Δ [*SIS1*, *URA3*] pRS313-Sis1_1–121_ strain (Johnson and Craig 2001). Therefore, we tested the ability of *sis1*Δ*ydj1*Δ [*SIS1*, *URA3*] pRS313-Sis1_1–121_ cells expressing atDjA1 or atDjA2 to lose the *URA3*-marked Sis1 plasmid on 5-FOA plates. Growth on synthetic media containing 5-FOA counterselects against the *URA3*-marked plasmid and only the cells that stochastically lose the *URA3*-marked plasmid form colonies. Both atDjA1 and atDjA2 proteins behaved like full-length Ydj1 and allowed cells to lose the *URA3*-marked Sis1 plasmid (Figure 6B), suggesting that these two class I J proteins of *A. thaliana* possess Ydj1-like functions. Besides atDjA1 and atDjA2, atDjB1 (Sis1 ortholog) was the only other plant J protein that could rescue the lethality of *sis1*Δ*ydj1*Δ [*SIS1*, *URA3*] pRS313-Sis1_1–121_ on 5-FOA (Figure 6B). Data from yeast genetic studies indicate that atDjA1 and atDjA2 are functionally alike and might have redundant functions in *A. thaliana*.

Functional divergence in paralogs is also known to emerge from changes in gene regulation (Gabaldon and Koonin 2013). We reasoned that, although biochemically similar, atDjA1 and atDjA2 might be expressed differentially in *A. thaliana* resulting in regulatory subfunctionalization. To test this, we performed real-time quantitative PCR analysis. Transcripts of both atDjA1 and atDjA2 were uniformly expressed in all plant tissues analyzed, (Figure 6C); however, while the atDjA1 transcript was specifically induced by high temperature, that of atDjA2 was induced by all of the stresses including high temperature, low temperature, salt stress, and mannitol (osmotic stress), as well as mechanical injury (Figure 6C). These results suggest that atDjA1 and atDjA2, although very similar to each other, might have some specialized stress-associated functions in *A. thaliana*.

**Figure 7:**
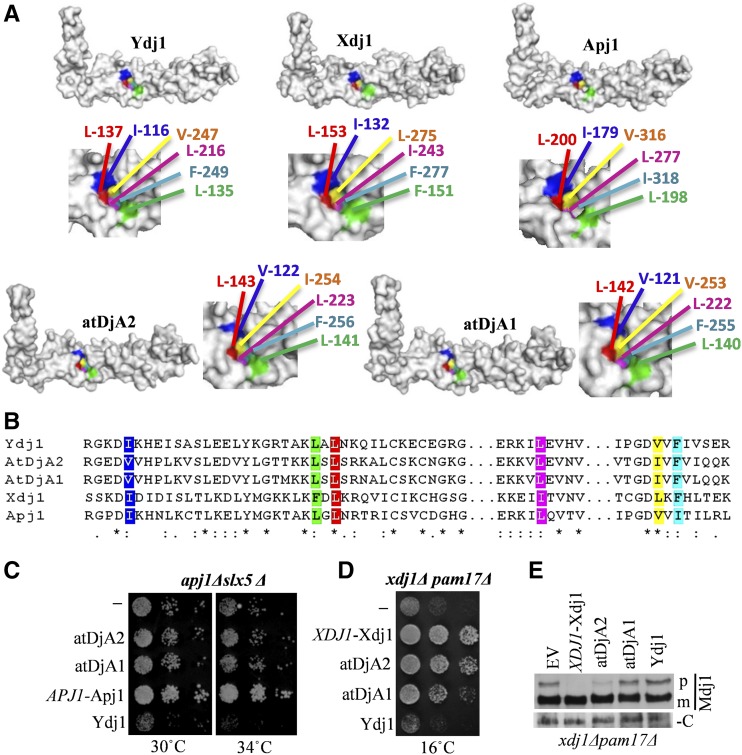
atDjA2 has Xdj1-like functions. (A) atDjA2 and atDjA1 have peptide-binding clefts similar to Ydj1, Xdj1, and Apj1. (Top) Surface-filled models of C-terminal region of Ydj1 (PDB:1NLT), Xdj1 (residues 64–316), Apj1 (residues 174 to 412), atDjA2 (residues 116–345), and atDjA1 (residues 115–344) were made using SWISS MODELER homology-modeling server. (Middle) Models of peptide-binding clefts of the indicated J proteins, based on the crystal structure of Ydj1. (B) Amino acid residues forming the peptide-binding cleft highlighted in the sequence alignment. (C) Five microliters from 10-fold serial dilutions of *apj1*Δ*slx5*Δ cells harboring either an empty pRS414-*TEF* vector (−) or different J protein expression plasmids [pRS314-Apj1 (*APJ1*-Apj1), pRS414-*TEF*-atDjA2 (atDjA2), pRS414-*TEF*-atDjA1 (atDjA1), or pRS414-*TEF*-Ydj1] were spotted on tryptophan drop-out (Trp DO) plates and incubated at indicated temperature for 3 d. (D) Five microliters from 10-fold serial dilutions of *xdj1*Δ*pam17*Δ cells harboring either an empty pRS413-*TEF* vector (−) or J protein expression plasmids [pRS313-Xdj1 (*XDJ1*-Xdj1), pRS413-*TEF*-atDjA2 (atDjA2), pRS413-*TEF*-atDjA1 (atDjA1), or pRS413-*TEF*-Ydj1] were spotted on histidine drop-out (His DO) plates and incubated at indicated temperature for 10 d. (E) Equal amounts of total cell lysate prepared from *xdj1*Δ*pam17*Δ cells either harboring an empty plasmid (−) or plasmids expressing wild-type Xdj1 (*XDJ1*-Xdj1), pRS413-*TEF*-atDjA2 (atDjA2), pRS413-*TEF*-atDjA1 (atDjA1), or pRS413-*TEF*-Ydj1 (Ydj1), were resolved on sodium dodecyl sulfate polyacrylamide gel electrophoresis, electroblotted on to a polyvinylidene fluoride membrane, probed with anti-Mdj1 antibody, and developed by chemiluminescence to show the cytosolic precursor form (p) and mature mitochondrial form (m) of mitochondrial protein Mdj1. Anti-RAP1 antibody was used as loading control (C).

In addition to Ydj1, which is conserved in higher eukaryotes, the *S. cerevisiae* cytosol has two more class I J proteins, Apj1 and Xdj1, which are highly specialized fungi-specific duplicates of Ydj1 (Sahi *et al.* 2013). The C-terminal CBDs of both of these J proteins share a very high degree of similarity to Ydj1, as well as Xdj1 and Apj1 (Figure 7A). Notably, even the hydrophobic client binding pocket is highly conserved among all five J proteins (Figure 7B). Therefore, we tested whether atDjA1 or atDjA2 could also replace Xdj1 or Apj1 in yeast. Although deletion of Apj1 in *S. cerevisiae* does not result in an observable phenotype, Apj1 exhibits a synthetic genetic interaction with Slx5, a SUMO-targeting ubiquitin ligase (STUbL), thus Apj1 has been implicated in protein degradation. *apj1*Δ*slx5*Δ cells are slow growing and exhibit temperature sensitivity (Sahi *et al.* 2013). *apj1*Δ*slx5*Δ cells expressing either atDjA1 or atDjA2 did not rescue the temperature sensitivity of the *apj1*Δ*slx5*Δ strain like wild-type Apj1 (Figure 7C). Moderate overexpression of Ydj1 also failed to rescue the growth defects of *apj1*Δ*slx5*Δ (Figure 7C), suggesting that the requirement for Apj1 in *S. cerevisiae* is unique and that the tested plant J proteins cannot substitute for yeast Apj1.

Next, we asked if atDjA1 and atDjA2 had Xdj1-like functions. Deletion of Xdj1 alone also has no obvious phenotype but exhibits a synthetic genetic interaction with Pam17, a component of the mitochondrial import motor, thus Xdj1 has been implicated in mitochondrial protein import in *S. cerevisiae* (Sahi *et al.* 2013). *xdj1*Δ*pam17*Δ double-knockout cells grow slowly at lower temperatures and exhibit mitochondrial import defects. This phenotype is specific to Xdj1, as the deletion of neither Apj1 nor Ydj1 shows a similar synthetic growth defect in a *pam17*Δ genetic background. The ability of atDjA1 and atDjA2 to rescue the growth and mitochondrial import defects of *xdj1*Δ*pam17*Δ was tested. While atDjA2 rescued the growth of *xdj1*Δ*pam17*Δ almost like Xdj1 (Figure 7D), atDjA1 could only partially rescue *xdj1*Δ*pam17*Δ at 16°. Furthermore, expression of Ydj1 did not alleviate the growth of the *xdj1*Δ*pam17*Δ strain (Figure 7D), indicating that although structurally similar, Xdj1 is highly specialized and functionally distinct from Ydj1. Several proteins that are destined to be imported into mitochondria bear a mitochondrial targeting signal sequence that is cleaved by specific proteases residing in the mitochondrial matrix. We used the accumulation of the mitochondrial J protein Mdj1 precursor to detect mitochondrial import defects. Wild-type yeast cells did not accumulate any cytosolic form of Mdj1; however, as reported previously, *xdj1*Δ*pam17*Δ cells showed accumulation of Mdj1 in its precursor form. This defect was significantly rescued by wild-type Xdj1 as well as atDjA2, but not by Ydj1 (Figure 7E). atDjA1 showed a subtle but reproducible reduction in the accumulation of the precursor form but was insignificant when compared to atDjA2 or Xdj1 (Figure 7E). Taken together, these data suggest that, besides carrying out generalized chaperone functions like Ydj1, atDjA2, and atDjA1 also have Xdj1-like functions and could be involved in mitochondrial protein import in *A. thaliana*.

## Discussion

J proteins are ubiquitous and arguably the most versatile chaperone proteins. With their obligate partners, Hsp70s, J proteins are involved in a wide array of cellular processes. In this study, we aimed to understand the emerging complexity of the J protein network and decipher how this is adding to the functional plasticity of the Hsp70:J protein chaperone machines in higher plants. The expanding multiplicity and functional diversity of J proteins in plants can facilitate the emergence of novel chaperone functions or the modification and fine-tuning of existing ones, thus allowing unprecedented versatility and specificity of the Hsp70:J protein machinery in higher plants.

Based on the functional studies employing yeast genetics, we conclude that cytosolic J protein complement involved in fundamental chaperone functions like translation initiation, ribosome biogenesis, folding of the nascent polypeptides, DPH biosynthesis, folding of cytosolic proteins, clathrin uncoating, and the remodeling of protein aggregates are highly conserved between *S. cerevisiae* and *A. thaliana*. Because the involvement of J proteins in cytosolic protein folding, the remodelling of protein aggregates, and clathrin uncoating has proliferated in *Arabidopsis*, this hints at a staggering complexity of cytosolic protein quality control and trafficking processes in higher plants. Additionally, the emergence of novel J proteins in *A. thaliana* might be contributing to the expansion of the cellular chaperome catering to plant-specific requirements. Moreover, it might also be increasing the cooperativity or redundancy between J proteins, further expanding the multiplicity of the Hsp70:J protein networks in plants.

The fact that plant J proteins were able to substitute for their corresponding orthologs in *S. cerevisiae* confirms that the Hsp70:J protein interaction is evolutionarily conserved. This further highlights the munificent nature of the ATPase domain of the multifunctional Hsp70, Ssa, of the yeast cytosol. Although the J domain is critical for J protein function, its contribution toward determining the specificity of an Hsp70:J protein machine is negligible (Kampinga and Craig 2010). Regions outside the J domain are often engaged in client binding or targeting of the J protein to a particular subcellular localization, thereby determining the specificity of a J protein. Most of the plant J proteins maintained the same specificities as their budding yeast orthologs. Consistent with this, like Jjj1, atDjC12 also interacts with 60S ribosomal maturation factors (Demoinet *et al.* 2007; Schmidt *et al.* 2014). In addition, the ability of atDjC12 to partially substitute for Zuo1 when overexpressed (Albanese *et al.* 2010; Meyer *et al.* 2007) signifies that atDjC12 can not only interact with preribosomal factors, but can also possibly operate with the multi-functional Hsp70, Ssa, and fold nascent polypeptides emerging from the ribosome. Along with the generalized Hsp70s of the *Arabidopsis* cytosol, atDjC12 could be involved ribosomal biogenesis and cotranslational protein folding. Another J protein involved with the *S. cerevisiae* translation machinery is Jjj3, which is essential for DPH biosynthesis (Liu *et al.* 2004). Although the physiological role of DPH biosynthesis is not clear, it is believed to have an important role in translation under normal and stress conditions (Arguelles *et al.* 2014; Schaffrath *et al.* 2014). While deletion of Jjj3 does not result in any observable growth defects in *S. cerevisiae*, mutations in the mouse ortholog *DPH4* causes developmental defects and lethality (Webb *et al.* 2008). It is possible that, in addition to their conserved role in DPH biosynthesis, orthologs of Jjj3 in higher eukaryotes might perform additional moonlighting functions. As a case in point, novel iron-mediated redox and electron carrier activity is present in the human orthologs *DPH4* (Thakur *et al.* 2012) and atDjC13 (A. K. Verma and C. Sahi, unpublished data), but not in Jjj3 of *S. cerevisiae* (Dong *et al.* 2014). It is possible that atDjC13 sequesters iron and is a regulator of intracellular iron homeostasis in *A. thaliana*, or that it has iron-mediated redox and electron carrier activity.

Clathrin-mediated endocytosis is an evolutionary conserved process. It controls turnover of plasma membrane proteins and cell wall biosynthesis enzymes, thus regulating different processes in eukaryotic cells (Fan *et al.* 2015; McMahon and Boucrot 2011). The J protein Swa2 recruits Hsp70 to clathrin cages, which removes the clathrin coats after endocytosis (Sousa and Lafer 2015). The ability of atDjC10 to rescue the growth defects of *swa2*Δ in a J domain-dependent manner suggests that, like Swa2, atDjC10 could be partnering with cytosolic Hsp70s to facilitate clathrin-mediated endocytosis in *Arabidopsis*. Further, six additional orthologs of Swa2 were predicted in *A. thaliana*, which underlines the importance and emerging complexity of clathrin-mediated endocytosis in higher plants. It is possible that different members of this group could have redundant or specialized roles in modulating clathrin dynamics.

Along with the disaggregase Hsp104 and the Hsp70 Ssa, Sis1 functions in an essential capacity for the fragmentation and, thus, propagation of all prions in yeast, potentially acting as the targeting factor directing the chaperone machinery to amyloids (Aron *et al.* 2007; Tipton *et al.* 2008). The fact that atDjB1 could substitute for Sis1 to maintain cell viability and at least two prions indicates a significant conservation of function. Although a definitive prion in plants has yet to be identified, numerous plant proteins important for flowering as well as growth and development have been found to have extended poly glutamine repeats that may support prion formation (Lindqvist *et al.* 2007; Rival *et al.* 2014; Undurraga *et al.* 2012). One such protein from *Arabidopsis*, Luminidependens (*LD*), was recently shown to fully function as a prion when expressed in *S. cerevisiae* (Chakrabortee *et al.* 2016). Given that plants are unique among higher eukaryotes in that they possess a homolog of the yeast Hsp100 protein critical to yeast prion propagation, as well as decimation of stress induced and polyglutamine (polyQ) aggregates (Mishra and Grover 2015; Sweeny and Shorter 2015), our results strongly suggest that plants might utilize a similar but increasingly complex J protein:Hsp70:Hsp100 machine, with atDjB1 or other Sis1 orthologs plausibly acting as the J protein-targeting factor for remodeling protein aggregates, thereby affecting plant growth and development in addition to stress tolerance in *Arabidopsis*.

The major cytosolic J protein Ydj1 is ubiquitous and functionally conserved in eukaryotes (Sarkar *et al.* 2013; Terada *et al.* 1997). As expected, both atDjA1 and atDjA2 were able to functionally compensate for Ydj1, suggesting that atDjA1 and atDjA2 are generalized J proteins of the *Arabidopsis* cytosol, like Ydj1 in *S. cerevisiae*. Consistent with this, atDjA1 and atDjA2 have been shown to have redundant stress-associated functions in *A. thaliana* (Li *et al.* 2007). Besides Ydj1, there are two more structurally similar class I J proteins in the cytosol of *S. cerevisiae*, Apj1 and Xdj1. The inability of atDjA1 and atDjA2 to substitute for Apj1 is not surprising, and is an experimental confirmation of our previous report that Apj1 is restricted to only some fungi and is not found in other eukaryotes (Sahi *et al.* 2013). Interestingly, the synthetic deletion phenotype of Xdj1, which is not rescued by even Ydj1, was significantly rescued by atDjA2 and partially by atDjA1. This suggests that, in the absence of an Xdj1 ortholog in *A. thaliana*, the Ydj1 orthologs atDjA1 and atDjA2 have additional Xdj1-like activities. It is likely that subtle changes in the CBD of Ydj1 orthologs enabled it to recognize alternative substrates and that this promiscuity modified a preexisting activity, thus driving the functional diversification observed in atDjA1 and atDjA2 (Camps *et al.* 2007). Alternatively, it is also possible that atDjA1 and atDjA2 have emerged from an ancestral *YDJ1* gene and acquired new functions, a classic case of neofunctionalization (Rastogi and Liberles 2005). The observed difference in the gene expression profiles might define the functional specificity and differential requirements of atDjA1 and atDjA2 in modulating protein quality control in the *Arabidopsis* cytosol under stress conditions.

Higher expression levels and client promiscuity puts additional evolutionary constraints on proteins, thus proteins that are more abundant or have multiple protein substrates almost always exhibit slower evolutionary rates (Zhang and Yang 2015). As compared to Jjj1, Jjj3, Swa2, and Cwc23, both Ydj1 and Sis1 are more abundant in *S. cerevisiae* (Ghaemmaghami *et al.* 2003) and possibly other eukaryotes as well. Moreover, both Ydj1 and Sis1, which perform a myriad of cellular functions, are involved in a greater number of protein–protein interactions compared to other J proteins analyzed in this study (Gong *et al.* 2009), further supporting their slower evolutionary rates. Even with significant sequence divergence, Jjj1, Jjj3, and Swa2 orthologs retained the cellular functions of their yeast counterparts, possibly because of structural similarities between the yeast and plant orthologs. This is consistent with the fact that, compared to the primary amino acid sequence, tertiary structures of homologous proteins are often more conserved (Worth *et al.* 2009). Cwc23 was an exception as unlike other J protein orthologs, atDjC37 failed to complement the *cwc23*Δ strain. Cwc23 is the only J protein in *S. cerevisiae* whose essential function is distinct from its role as a J protein (Pandit *et al.* 2009; Sahi *et al.* 2010). Instead the C-terminus, which is important for interaction with spliceosomal factors, is required for proper growth and RNA splicing (Sahi *et al.* 2010). Emergence of functional differences between orthologs is, at times, much greater than expected, especially for low abundant proteins and proteins with fewer interactions like Cwc23 (Gabaldon and Koonin 2013; Ghaemmaghami *et al.* 2003; Gong *et al.* 2009). Although it is possible that atDjC37 has neofunctionalized in *A. thaliana*, it is also possible that atDjC37 is involved in complex molecular machinery carrying out RNA splicing with numerous coevolved protein–protein interactions that prevented the maintenance of function over a long evolutionary timescale.

In summary, we propose that the increasing number, regulatory differences, and acquisition of functional novelty by evolutionary tinkering of biochemical activities caused by some of the J proteins is expanding the J protein network in *A. thaliana*. Using yeast genetics, we linked specific plant J proteins to specific chaperone functions; however, these need to be validated in plant models as well. With seven Hsp70s in the *A. thaliana* cytosol, highly complex and possibly combinatorial Hsp70:J protein networks are likely to provide additional flexibility and robustness to cope with the increased biochemical complexity of chaperone functions in higher plants.

## Supplementary Material

Supplemental material is available online at www.g3journal.org/lookup/suppl/doi:10.1534/g3.117.042291/-/DC1.

## References

[bib1] Albanese V. Reissmann S. Frydman J., 2010 A ribosome-anchored chaperone network that facilitates eukaryotic ribosome biogenesis. J. Cell Biol. 189(1): 69–81. 20368619 10.1083/jcb.201001054 PMC2854368

[bib2] Arguelles S. Camandola S. Cutler R. G. Ayala A. Mattson M. P., 2014 Elongation factor 2 diphthamide is critical for translation of two IRES-dependent protein targets, XIAP and FGF2, under oxidative stress conditions. Free Radic. Biol. Med. 67: 131–138. 24140707 10.1016/j.freeradbiomed.2013.10.015 PMC3945166

[bib3] Arndt K. T. Styles C. A. Fink G. R., 1989 A suppressor of a HIS4 transcriptional defect encodes a protein with homology to the catalytic subunit of protein phosphatases. Cell 56(4): 527–537. 2537149 10.1016/0092-8674(89)90576-x

[bib4] Arnold K. Bordoli L. Kopp J. Schwede T., 2006 The SWISS-MODEL workspace: a web-based environment for protein structure homology modelling. Bioinformatics 22(2): 195–201. 16301204 10.1093/bioinformatics/bti770

[bib5] Aron R. Higurashi T. Sahi C. Craig E. A., 2007 J-protein co-chaperone Sis1 required for generation of [RNQ^+^] seeds necessary for prion propagation. EMBO J. 26(16): 3794–3803. 17673909 10.1038/sj.emboj.7601811 PMC1952226

[bib6] Bekh-Ochir D. Shimada S. Yamagami A. Kanda S. Ogawa K., 2013 A novel mitochondrial DnaJ/Hsp40 family protein BIL2 promotes plant growth and resistance against environmental stress in brassinosteroid signaling. Planta 237(6): 1509–1525. 23494613 10.1007/s00425-013-1859-3 PMC3664749

[bib7] Boorstein W. R. Ziegelhoffer T. Craig E. A., 1994 Molecular evolution of the HSP70 multigene family. J. Mol. Evol. 38(1): 1–17. 8151709 10.1007/BF00175490

[bib8] Boston R. S. Viitanen P. V. Vierling E., 1996 Molecular chaperones and protein folding in plants. Plant Mol. Biol. 32(1–2): 191–222. 8980480 10.1007/BF00039383

[bib9] Bukau B. Horwich A. L., 1998 The Hsp70 and Hsp60 chaperone machines. Cell 92(3): 351–366. 9476895 10.1016/s0092-8674(00)80928-9

[bib10] Bukau B. Weissman J. Horwich A., 2006 Molecular chaperones and protein quality control. Cell 125(3): 443–451. 16678092 10.1016/j.cell.2006.04.014

[bib11] Bustin S. A. Benes V. Garson J. A. Hellemans J. Huggett J., 2009 The MIQE guidelines: minimum information for publication of quantitative real-time PCR experiments. Clin. Chem. 55(4): 611–622. 19246619 10.1373/clinchem.2008.112797

[bib12] Camps M. Herman A. Loh E. Loeb L. A., 2007 Genetic constraints on protein evolution. Crit. Rev. Biochem. Mol. Biol. 42(5): 313–326. 17917869 10.1080/10409230701597642 PMC3825456

[bib13] Chakrabortee S. Kayatekin C. Newby G. A. Mendillo M. L. Lancaster A., 2016 Luminidependens (LD) is an Arabidopsis protein with prion behavior. Proc. Natl. Acad. Sci. USA 113(21): 6065–6070. 27114519 10.1073/pnas.1604478113 PMC4889399

[bib14] Cheetham M. E. Caplan A. J., 1998 Structure, function and evolution of DnaJ: conservation and adaptation of chaperone function. Cell Stress Chaperones 3(1): 28–36. 9585179 10.1379/1466-1268(1998)003<0028:sfaeod>2.3.co;2 PMC312945

[bib15] Chen J. Y. Bodley J. W. Livingston D. M., 1985 Diphtheria toxin-resistant mutants of Saccharomyces cerevisiae. Mol. Cell. Biol. 5(12): 3357–3360. 3915773 10.1128/mcb.5.12.3357-3360.1985 PMC369163

[bib16] Czechowski T. Bari R. P. Stitt M. Scheible W. R. Udvardi M. K., 2004 Real-time RT-PCR profiling of over 1400 Arabidopsis transcription factors: unprecedented sensitivity reveals novel root- and shoot-specific genes. Plant J. 38(2): 366–379. 15078338 10.1111/j.1365-313X.2004.02051.x

[bib17] Demoinet E. Jacquier A. Lutfalla G. Fromont-Racine M., 2007 The Hsp40 chaperone Jjj1 is required for the nucleo-cytoplasmic recycling of preribosomal factors in Saccharomyces cerevisiae. RNA 13(9): 1570–1581. 17652132 10.1261/rna.585007 PMC1950757

[bib18] Dong M. Su X. Dzikovski B. Dando E. E. Zhu X., 2014 Dph3 is an electron donor for Dph1-Dph2 in the first step of eukaryotic diphthamide biosynthesis. J. Am. Chem. Soc. 136(5): 1754–1757. 24422557 10.1021/ja4118957 PMC3985478

[bib19] Dorn K. V. Willmund F. Schwarz C. Henselmann C. Pohl T., 2010 Chloroplast DnaJ-like proteins 3 and 4 (CDJ3/4) from Chlamydomonas reinhardtii contain redox-active Fe-S clusters and interact with stromal HSP70B. Biochem. J. 427(2): 205–215. 20113313 10.1042/BJ20091412

[bib20] Fan L. Li R. Pan J. Ding Z. Lin J., 2015 Endocytosis and its regulation in plants. Trends Plant Sci. 20(6): 388–397. 25914086 10.1016/j.tplants.2015.03.014

[bib21] Gabaldon T. Koonin E. V., 2013 Functional and evolutionary implications of gene orthology. Nat. Rev. Genet. 14(5): 360–366. 23552219 10.1038/nrg3456 PMC5877793

[bib22] Ghaemmaghami S. Huh W. K. Bower K. Howson R. W. Belle A., 2003 Global analysis of protein expression in yeast. Nature 425(6959): 737–741. 14562106 10.1038/nature02046

[bib23] Gong Y. Kakihara Y. Krogan N. Greenblatt J. Emili A., 2009 An atlas of chaperone-protein interactions in Saccharomyces cerevisiae: implications to protein folding pathways in the cell. Mol. Syst. Biol. 5: 275. 19536198 10.1038/msb.2009.26 PMC2710862

[bib24] Harris J. M. Nguyen P. P. Patel M. J. Sporn Z. A. Hines J. K., 2014 Functional diversification of hsp40: distinct j-protein functional requirements for two prions allow for chaperone-dependent prion selection. PLoS Genet. 10(7): e1004510. 25058638 10.1371/journal.pgen.1004510 PMC4109904

[bib25] Hartl F. U. Bracher A. Hayer-Hartl M., 2011 Molecular chaperones in protein folding and proteostasis. Nature 475(7356): 324–332. 21776078 10.1038/nature10317

[bib26] Higurashi T. Hines J. K. Sahi C. Aron R. Craig E. A., 2008 Specificity of the J-protein Sis1 in the propagation of 3 yeast prions. Proc. Natl. Acad. Sci. USA 105(43): 16596–16601. 18955697 10.1073/pnas.0808934105 PMC2575465

[bib27] Hines J. K. Craig E. A., 2011 The sensitive [SWI (+)] prion: new perspectives on yeast prion diversity. Prion 5(3): 164–168. 21811098 10.4161/pri.5.3.16895 PMC3226042

[bib28] Hines J. K. Li X. Du Z. Higurashi T. Li L., 2011 [SWI], the prion formed by the chromatin remodeling factor Swi1, is highly sensitive to alterations in Hsp70 chaperone system activity. PLoS Genet. 7(2): e1001309. 21379326 10.1371/journal.pgen.1001309 PMC3040656

[bib29] Huerta-Cepas J. Serra F. Bork P., 2016 ETE 3: reconstruction, analysis, and visualization of phylogenomic data. Mol. Biol. Evol. 33(6): 1635–1638. 26921390 10.1093/molbev/msw046 PMC4868116

[bib30] Johnson J. L. Craig E. A., 2001 An essential role for the substrate-binding region of Hsp40s in Saccharomyces cerevisiae. J. Cell Biol. 152(4): 851–856. 11266475 10.1083/jcb.152.4.851 PMC2195774

[bib31] Kampinga H. H. Craig E. A., 2010 The HSP70 chaperone machinery: J proteins as drivers of functional specificity. Nat. Rev. Mol. Cell Biol. 11(8): 579–592. 20651708 10.1038/nrm2941 PMC3003299

[bib32] Katoh K. Misawa K. Kuma K. Miyata T., 2002 MAFFT: a novel method for rapid multiple sequence alignment based on fast Fourier transform. Nucleic Acids Res. 30(14): 3059–3066. 12136088 10.1093/nar/gkf436 PMC135756

[bib33] Kong F. Deng Y. Zhou B. Wang G. Wang Y., 2014 A chloroplast-targeted DnaJ protein contributes to maintenance of photosystem II under chilling stress. J. Exp. Bot. 65(1): 143–158. 24227338 10.1093/jxb/ert357 PMC3883286

[bib34] Koonin E. V., 2005 Orthologs, paralogs, and evolutionary genomics. Annu. Rev. Genet. 39: 309–338. 16285863 10.1146/annurev.genet.39.073003.114725

[bib35] Kosova K. Vitamvas P. Prasil I. T. Renaut J., 2011 Plant proteome changes under abiotic stress–contribution of proteomics studies to understanding plant stress response. J. Proteomics 74(8): 1301–1322. 21329772 10.1016/j.jprot.2011.02.006

[bib36] Kryndushkin D. S. Alexandrov I. M. Ter-Avanesyan M. D. Kushnirov V. V., 2003 Yeast [PSI+] prion aggregates are formed by small Sup35 polymers fragmented by Hsp104. J. Biol. Chem. 278(49): 49636–49643. 14507919 10.1074/jbc.M307996200

[bib37] Kurepa J. Wang S. Li Y. Zaitlin D. Pierce A. J., 2009 Loss of 26S proteasome function leads to increased cell size and decreased cell number in Arabidopsis shoot organs. Plant Physiol. 150(1): 178–189. 19321709 10.1104/pp.109.135970 PMC2675745

[bib38] Lee D. Redfern O. Orengo C., 2007 Predicting protein function from sequence and structure. Nat. Rev. Mol. Cell Biol. 8(12): 995–1005. 18037900 10.1038/nrm2281

[bib39] Li G.-L. Chang H. Li B. Zhou W. Sun D.-Y., 2007 The roles of the atDjA2 and atDjA3 molecular chaperone proteins in improving thermotolerance of Arabidopsis thaliana seedlings. Plant Sci. 173(4): 408–416.

[bib40] Liebman S. W. Chernoff Y. O., 2012 Prions in yeast. Genetics 191: 1041–1072. 22879407 10.1534/genetics.111.137760 PMC3415993

[bib41] Lindqvist C. Laakkonen L. Albert V. A., 2007 Polyglutamine variation in a flowering time protein correlates with island age in a Hawaiian plant radiation. BMC Evol. Biol. 7: 105. 17605781 10.1186/1471-2148-7-105 PMC1939987

[bib42] Liu S. Milne G. T. Kuremsky J. G. Fink G. R. Leppla S. H., 2004 Identification of the proteins required for biosynthesis of diphthamide, the target of bacterial ADP-ribosylating toxins on translation elongation factor 2. Mol. Cell. Biol. 24(21): 9487–9497. 15485916 10.1128/MCB.24.21.9487-9497.2004 PMC522255

[bib43] Lopez N. Aron R. Craig E. A., 2003 Specificity of class II Hsp40 Sis1 in maintenance of yeast prion [RNQ^+^]. Mol. Biol. Cell 14(3): 1172–1181. 12631732 10.1091/mbc.E02-09-0593 PMC151588

[bib44] Lu S. Van Eck J. Zhou X. Lopez A. B. O’Halloran D. M., 2006 The cauliflower Or gene encodes a DnaJ cysteine-rich domain-containing protein that mediates high levels of beta-carotene accumulation. Plant Cell 18(12): 3594–3605. 17172359 10.1105/tpc.106.046417 PMC1785402

[bib45] Mattheakis L. C. Shen W. H. Collier R. J., 1992 DPH5, a methyltransferase gene required for diphthamide biosynthesis in Saccharomyces cerevisiae. Mol. Cell. Biol. 12(9): 4026–4037. 1508200 10.1128/mcb.12.9.4026-4037.1992 PMC360293

[bib46] McMahon H. T. Boucrot E., 2011 Molecular mechanism and physiological functions of clathrin-mediated endocytosis. Nat. Rev. Mol. Cell Biol. 12(8): 517–533. 21779028 10.1038/nrm3151

[bib47] Meyer A. E. Hung N. J. Yang P. Johnson A. W. Craig E. A., 2007 The specialized cytosolic J-protein, Jjj1, functions in 60S ribosomal subunit biogenesis. Proc. Natl. Acad. Sci. USA 104(5): 1558–1563. 17242366 10.1073/pnas.0610704104 PMC1785244

[bib48] Miernyk J. A., 1999 Protein folding in the plant cell. Plant Physiol. 121(3): 695–703. 10557217 10.1104/pp.121.3.695 PMC1539232

[bib49] Mishra R. C. Grover A., 2015 ClpB/Hsp100 proteins and heat stress tolerance in plants. Crit. Rev. Biotechnol. 36: 862–874. 26121931 10.3109/07388551.2015.1051942

[bib50] Mumberg D. Müller R. Funk M., 1995 Yeast vectors for the controlled expression of heterologous proteins in different genetic backgrounds. Gene 156(1): 119–122. 7737504 10.1016/0378-1119(95)00037-7

[bib51] Nover L. Miernyk J. A., 2001 A genomics approach to the chaperone network of Arabidopsis thaliana. Cell Stress Chaperones 6(3): 175–176. 11599558 10.1379/1466-1268(2001)006<0175:agattc>2.0.co;2 PMC434398

[bib52] Pandit S. Paul S. Zhang L. Chen M. Durbin N., 2009 Spp382p interacts with multiple yeast splicing factors, including possible regulators of Prp43 DExD/H-Box protein function. Genetics 183: 195–206. 19581443 10.1534/genetics.109.106955 PMC2746144

[bib53] Park S. H. Kukushkin Y. Gupta R. Chen T. Konagai A., 2013 PolyQ proteins interfere with nuclear degradation of cytosolic proteins by sequestering the Sis1p chaperone. Cell 154(1): 134–145. 23791384 10.1016/j.cell.2013.06.003

[bib54] Pishvaee B. Costaguta G. Yeung B. G. Ryazantsev S. Greener T., 2000 A yeast DNA J protein required for uncoating of clathrin-coated vesicles in vivo. Nat. Cell Biol. 2(12): 958–963. 11146663 10.1038/35046619

[bib55] Rajan V. B. D’Silva P., 2009 Arabidopsis thaliana J-class heat shock proteins: cellular stress sensors. Funct. Integr. Genomics 9(4): 433–446. 19633874 10.1007/s10142-009-0132-0

[bib56] Rastogi S. Liberles D. A., 2005 Subfunctionalization of duplicated genes as a transition state to neofunctionalization. BMC Evol. Biol. 5: 28. 15831095 10.1186/1471-2148-5-28 PMC1112588

[bib57] Reidy M. Sharma R. Shastry S. Roberts B.-L. Albino-Flores I., 2014 Hsp40s specify functions of Hsp104 and Hsp90 protein chaperone machines. PLoS Genet. 10(10): e1004720. 25329162 10.1371/journal.pgen.1004720 PMC4199505

[bib58] Rival P. Press M. O. Bale J. Grancharova T. Undurraga S. F., 2014 The conserved PFT1 tandem repeat is crucial for proper flowering in Arabidopsis thaliana. Genetics 198: 747–754. 25116137 10.1534/genetics.114.167866 PMC4196625

[bib59] Sahi C. Craig E. A., 2007 Network of general and specialty J protein chaperones of the yeast cytosol. Proc. Natl. Acad. Sci. USA 104(17): 7163–7168. 17438278 10.1073/pnas.0702357104 PMC1855418

[bib60] Sahi C. Lee T. Inada M. Pleiss J. A. Craig E. A., 2010 Cwc23, an essential J protein critical for pre-mRNA splicing with a dispensable J domain. Mol. Cell. Biol. 30(1): 33–42. 19822657 10.1128/MCB.00842-09 PMC2798280

[bib61] Sahi C. Kominek J. Ziegelhoffer T. Yu H. Y. Baranowski M., 2013 Sequential duplications of an ancient member of the DnaJ-family expanded the functional chaperone network in the eukaryotic cytosol. Mol. Biol. Evol. 30(5): 985–998. 23329686 10.1093/molbev/mst008 PMC3670730

[bib62] Sarkar N. K. Thapar U. Kundnani P. Panwar P. Grover A., 2013 Functional relevance of J-protein family of rice (Oryza sativa). Cell Stress Chaperones 18(3): 321–331. 23160806 10.1007/s12192-012-0384-9 PMC3631087

[bib63] Schaffrath R. Abdel-Fattah W. Klassen R. Stark M. J., 2014 The diphthamide modification pathway from Saccharomyces cerevisiae–revisited. Mol. Microbiol. 94(6): 1213–1226. 25352115 10.1111/mmi.12845

[bib64] Schmidt S. Dethloff F. Beine-Golovchuk O. Kopka J., 2014 REIL proteins of Arabidopsis thaliana interact in yeast-2-hybrid assays with homologs of the yeast Rlp24, Rpl24A, Rlp24B, Arx1, and Jjj1 proteins. Plant Signal. Behav. 9(3): e28224. 24603461 10.4161/psb.28224 PMC4091606

[bib65] Sondheimer N. Lopez N. Craig E. A. Lindquist S., 2001 The role of Sis1 in the maintenance of the [RNQ^+^] prion. EMBO J. 20(10): 2435–2442. 11350932 10.1093/emboj/20.10.2435 PMC125465

[bib66] Sousa R. Lafer E. M., 2015 The role of molecular chaperones in clathrin mediated vesicular trafficking. Front. Mol. Biosci. 2: 26. 26042225 10.3389/fmolb.2015.00026 PMC4436892

[bib67] Sporn Z. A. Hines J. K., 2015 Hsp40 function in yeast prion propagation: amyloid diversity necessitates chaperone functional complexity. Prion 9(2): 80–89. 25738774 10.1080/19336896.2015.1020268 PMC4601347

[bib68] Summers D. W. Wolfe K. J. Ren H. Y. Cyr D. M., 2013 The Type II Hsp40 Sis1 cooperates with Hsp70 and the E3 ligase Ubr1 to promote degradation of terminally misfolded cytosolic protein. PLoS One 8(1): e52099. 23341891 10.1371/journal.pone.0052099 PMC3547041

[bib69] Sung D. Y. Vierling E. Guy C. L., 2001 Comprehensive expression profile analysis of the Arabidopsis Hsp70 gene family. Plant Physiol. 126(2): 789–800. 11402207 10.1104/pp.126.2.789 PMC111169

[bib70] Sweeny E. A. Shorter J., 2015 Mechanistic and structural insights into the prion-disaggregase activity of Hsp104. J. Mol. Biol. 428: 1870–1885. 26608812 10.1016/j.jmb.2015.11.016 PMC4860052

[bib71] Tanaka M. Collins S. R. Toyama B. H. Weissman J. S., 2006 The physical basis of how prion conformations determine strain phenotypes. Nature 442(7102): 585–589. 16810177 10.1038/nature04922

[bib72] Terada K. Kanazawa M. Bukau B. Mori M., 1997 The human DnaJ homologue dj2 facilitates mitochondrial protein import and luciferase refolding. J. Cell Biol. 139(5): 1089–1095. 9382858 10.1083/jcb.139.5.1089 PMC2140199

[bib73] Thakur A. Chitoor B. Goswami A. V. Pareek G. Atreya H. S., 2012 Structure and mechanistic insights into novel iron-mediated moonlighting functions of human J-protein cochaperone, Dph4. J. Biol. Chem. 287(16): 13194–13205. 22367199 10.1074/jbc.M112.339655 PMC3339945

[bib74] Thomas B. J. Rothstein R., 1989 The genetic control of direct-repeat recombination in Saccharomyces: the effect of rad52 and rad1 on mitotic recombination at GAL10, a transcriptionally regulated gene. Genetics 123: 725–738. 2693208 10.1093/genetics/123.4.725 PMC1203884

[bib75] Tipton K. A. Verges K. J. Weissman J. S., 2008 In vivo monitoring of the prion replication cycle reveals a critical role for Sis1 in delivering substrates to Hsp104. Mol. Cell 32(4): 584–591. 19026788 10.1016/j.molcel.2008.11.003 PMC2875781

[bib76] Torgerson D. G. Singh R. S., 2004 Rapid evolution through gene duplication and subfunctionalization of the testes-specific α4 proteasome subunits in Drosophila. Genetics 168: 1421–1432. 15579695 10.1534/genetics.104.027631 PMC1448786

[bib77] Troisi E. M. Rockman M. E. Nguyen P. P. Oliver E. E. Hines J. K., 2015 Swa2, the yeast homolog of mammalian auxilin, is specifically required for the propagation of the prion variant [URE3–1]. Mol. Microbiol. 97(5): 926–941. 26031938 10.1111/mmi.13076 PMC4689296

[bib78] Undurraga S. F. Press M. O. Legendre M. Bujdoso N. Bale J., 2012 Background-dependent effects of polyglutamine variation in the Arabidopsis thaliana gene ELF3. Proc. Natl. Acad. Sci. USA 109(47): 19363–19367. 23129635 10.1073/pnas.1211021109 PMC3511081

[bib79] Walsh P. Bursać D. Law Y. C. Cyr D. Lithgow T., 2004 The J-protein family: modulating protein assembly, disassembly and translocation. EMBO Rep. 5(6): 567–571. 15170475 10.1038/sj.embor.7400172 PMC1299080

[bib80] Wang G. Kong F. Zhang S. Meng X. Wang Y., 2015 A tomato chloroplast-targeted DnaJ protein protects Rubisco activity under heat stress. J. Exp. Bot. 66(11): 3027–3040. 25801077 10.1093/jxb/erv102

[bib81] Wang W. Vinocur B. Shoseyov O. Altman A., 2004 Role of plant heat-shock proteins and molecular chaperones in the abiotic stress response. Trends Plant Sci. 9(5): 244–252. 15130550 10.1016/j.tplants.2004.03.006

[bib82] Webb T. R. Cross S. H. McKie L. Edgar R. Vizor L., 2008 Diphthamide modification of EEF2 requires a J-domain protein and is essential for normal development. J. Cell Sci. 121(Pt. 19): 3140–3145. 18765564 10.1242/jcs.035550 PMC2592597

[bib83] Worth C. L. Gong S. Blundell T. L., 2009 Structural and functional constraints in the evolution of protein families. Nat. Rev. Mol. Cell Biol. 10(10): 709–720. 19756040 10.1038/nrm2762

[bib84] Xiao J. Kim L. S. Graham T. R., 2006 Dissection of Swa2p/auxilin domain requirements for cochaperoning Hsp70 clathrin-uncoating activity in vivo. Mol. Biol. Cell 17(7): 3281–3290. 16687570 10.1091/mbc.E06-02-0106 PMC1483056

[bib85] Yan W. Craig E. A., 1999 The glycine-phenylalanine-rich region determines the specificity of the yeast Hsp40 Sis1. Mol. Cell. Biol. 19(11): 7751–7758. 10523664 10.1128/mcb.19.11.7751 PMC84827

[bib86] Yang J. H. Huai Y. Zhang M. F., 2009 Mitochondrial atpA gene is altered in a new orf220-type cytoplasmic male-sterile line of stem mustard (Brassica juncea). Mol. Biol. Rep. 36(2): 273–280. 18026850 10.1007/s11033-007-9176-1

[bib87] Yang Y. Qin Y. Xie C. Zhao F. Zhao J., 2010 The Arabidopsis chaperone J3 regulates the plasma membrane H^+^-ATPase through interaction with the PKS5 kinase. Plant Cell 22(4): 1313–1332. 20418496 10.1105/tpc.109.069609 PMC2879748

[bib88] Zhang J. Yang J.-R., 2015 Determinants of the rate of protein sequence evolution. Nat. Rev. Genet. 16(7): 409–420. 26055156 10.1038/nrg3950 PMC4523088

