# Supplementary material for: Evolutionary Conservation and Emerging Functional Diversity of the Cytosolic Hsp70:J Protein Chaperone Network of Arabidopsis thaliana
Source: G3 (Bethesda). 2017 Apr 21;7(6):1941–54. doi: 10.1534/g3.117.042291 (PMC5473770; doi:10.1534/g3.117.042291)
Supplement: Supplementary file 1 [file 1941File001.pdf]

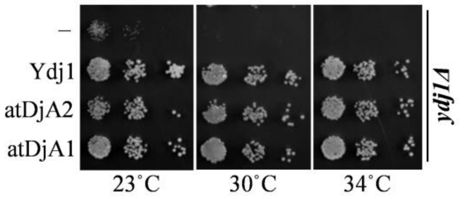

Figure S1. Complementation of *ydj1Δ* by atDjA2 and atDjA1. 5 µl of ten-fold serial dilutions of *ydj1Δ* transformed with either empty pRS413 plasmid (-) or, J protein expressing plasmids; pRS413-*TEF*-Ydj1, pRS413-*TEF*-atDjA2 and pRS413-*TEF*-atDjA1 on His drop-out plate and incubated at indicated temperatures.

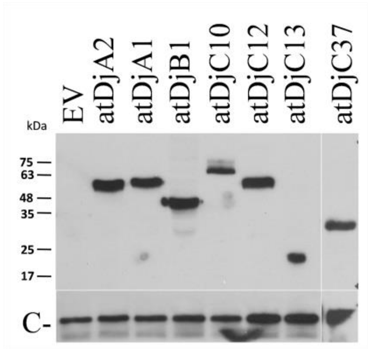

Figure S2. Expression levels of *A. thaliana* J protein in yeast cells. Equal amounts of total cell lysate prepared from wild-type cells either harboring an empty plasmid (-) or HA-tagged constructs pRS413-*TEF*-atDjA2 (atDjA2), pRS413-*TEF*-atDjA1 (atDjA1), pRS413-*TEF*-atDjB1 (atDjB1), pRS413-*TEF*-atDjC10 (atDjC10), pRS413-*TEF*-atDjC12 (atDjC12), pRS413-*TEF*-atDjC13 (atDjC13) and pRS413-*TEF*-atDjC37 (atDjC37), were resolved on SDS-PAGE, electro-blotted on to PVDF membrane, probed with anti-HA antibody, and developed by chemiluminescence. Anti-RAP1 antibody was used as loading control (C).

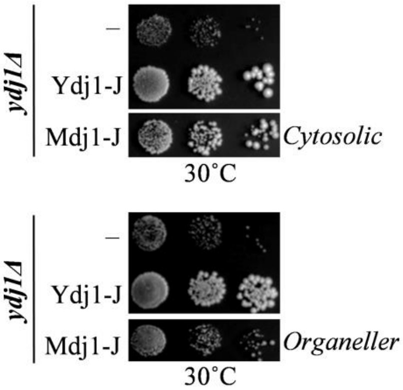

Figure S3. Complementation of *ydj1Δ* by Mdj1 J fragments. 5 μl of ten-fold serial dilutions of *ydj1Δ* transformed with either empty pRS414 plasmid (-) or, J domain fragment expressing plasmids; pRS414-*TEF*-Ydj1$_{1\text{-}220}$ (Ydj1-J), pRS414-*TEF*-Mdj1$_{56\text{-}186}$ (cytosolic Mdj1-J), pRS414-*TEF*-Mdj1$_{1\text{-}186}$ (organellar Mdj1-J) were spotted on Trp drop-out plates and incubated at 30°C for three days.

Table S1. List of primers used in this study.

Name	Sequence
*Sc*Ydj1_F	TAGTGGATCCATGGTTAAAGAAACTAAG
*Sc*Ydj1_R	GCTTATCGATTCATTGAGATGCACATTG
*Sc*Ydj1-J_R (1-220)	GCTTATCGATGACATGGACTTCTAGGATC
*Sc*Jjj3_F	ATCCACTAGTATGTCATTGGTGAATTCG
*Sc*Jjj3_R	GCTTATCGATTTATTGTCCTTCTTGCTCTTC
*Sc*Cwc23_F	ATCCACTAGTATGCCAGGACACGAATTGGAAGAC
*Sc*Cwc23_R	GCTTATCGATCTAGTCGAGTAGGATAGGTTC
atDjA2_F	ATCCACTAGTATGTTTGGAAGAGGACCTTC
atDjA2_R	GCTTATCGATTCACTGCTGGGCACATTG
atDjA1_F	ATCCACTAGTATGTTCGGTAGAGGAC
atDjA1_R	GCTTATCGATTTACTGCTGGGCACATTG
atDjB1_F	ATCCACTAGTATGGGGGTTGATTTCTATAAG
atDjB1_R	GCTTATCGATTCAAGGTGATAACATCCGCTTG
atDjC10 _F	ATCCACTAGTATGGACGAGTTTGGTGTTCTAACG
atDjC10 _R	GCTTATCGATTTAAGAGAGCTCCTCC
atDjC12 _F	TAGTGGATCCATGGCGTCTTCGTCTCGTTCG
atDjC12 _R	GAATTCTCATCGGGATTTCACTGTCG
atDjC13 _F	ATCCACTAGTATGCTTGTCGGTGAGAATTGTGTTCACG
atDjC13 _R	GCTTATCGATCTAGATAGGAATCTTCATATCAGAATCAACC
atDjA2:1-128_R/atDjA1:1-127_R	CCGGAATTCACCTTCAAGGGATGAACAAC
atDjA2:H42Q_F	AGCCGCTATCAAAAACCAGCCTGATAAGGGTGGT
atDjA2:H42Q_R	ACCACCCTTATCAGGCTGGTTTTTGATAGCGGCT
atDjA1:H42Q_F	AAGCCGCTATCAAGAATCAGCCTGATAAGGGTG
atDjA1:H42Q_R	CACCCTTATCAGGCTGATTCTTGATAGCGGCTT
atDjB1:H32Q_F	CAAACTCGCCATGAAATGGCAGCCTGATAAGAACC
atDjB1:H32Q_R	CCATTTCATGGCGAGTTTGCGAGTTTGCGAT
atDjC13:H39Q_F	CTCGTGTTTTCAAAGAATAAGAACCACAAGGAAGAACAACAG
atDjC13:H39Q_R	CTGTTGTTCTTCCTTGTGGTTCTTATTCTTTGAAAACACGAG
atDjC37_F	ATCCACTAGTATGGAGGGTTTTGTCG
atDjC37_R	GCTTATCGATTTAAACCCCTCGCTTTTTCTGG
Mdj1:1-186_F	ATCCACTAGTATGGCTTTCCAACAAG
Mdj1:1-186_R	GCTTATCGATTTAGCCACCGCTAC
Mdj1:56-186_F	ATCCACTAGTATGAACGAAGCATTCAAG
atDjA2_HA_F	ATCACTAGTATGTACCCATACGATGTTCCAGATTACGCTTTTGGAAGAG
atDjA1_HA_F	ATCACTAGTATGTACCCATACGATGTTCCAGATTACGCTTTCGGTAGAG
atDjC10 _HA_F	ATCACTAGTATGTACCCATACGATGTTCCAGATTACGCTGACGAGTTTG
atDjC12 _HA_F	ATCACTAGTATGTACCCATACGATGTTCCAGATTACGCTGCGTCTTCGT
atDjC13 _HA_F	ATCACTAGTATGTACCCATACGATGTTCCAGATTACGCTCTTGTCGGTG
atDjC37 _HA_F	ATCACTAGTATGTACCCATACGATGTTCCAGATTACGCTGAGGGTTTTG
atDjA2:rt_F	CCTTGTGTGGCTTCCAGTTT
atDjA2:rt_R	CGTGAAGTGAATGTATAGCTTACCC
atDjA1:rt_F	AGCTTTGTGTGGCTTCCAAT
atDjA1:rt_R	TGAAGTGGATGTAGAGCTTACCC
at_Actin_rt_F	GCCATCCAAGCTGTTCTCTC
at_Actin_rt_R	GCATGAGGAAGAGAGAAACCC

Table S2. ***Arabidopsis thaliana*** **J proteins.**

Si. No.	Gene Locus	Standard Name	Class	Protein size	J domain	Localization	Homolog
1	AT5G22060	atDjA2	I	419	13 to 67	Cyt.	Ydj1
2	AT3G44110	atDjA1	I	420	13 to 67	Cyt.	Ydj1
3	AT4G28480	atDjB2	II	348	3 to 62	Cyt.	Sis1
4	AT2G20560	atDjB3	II	337	3 to 62	Cyt.	Sis1
5	AT5G25530	atDjB10	II	347	3 to 70	Cyt.	Sis1
6	AT1G59725	atDjB6	II	331	3 to 63	Cyt.	Sis1
7	AT1G21080	atDjB19	II	400	5 to 63	Cyt.	Caj1
8	AT1G76700	atDjB8	II	398	5 to 63	Cyt.	Caj1
9	AT2G21510	atDjB20	II	346	5 to 63	Cyt.	Djp1
10	AT4G39150	atDjB7	II	345	5 to 63	Cyt.	Djp1
11	AT1G68370	atDjB11	II	410	16 to 74	Cyt.	-
12	AT3G47940	atDjB17	II	350	3 to 63	Cyt.	Sis1
13	AT5G49060	atDjC38	II	354	98 to 155	Cyt.	Hlj1
14	AT1G24120	atDjB12	II	436	19 to 77	Cyt.	-
15	AT5G49580	atDjB16	II	695	407 to 467	Cyt.	-
16	AT5G01390	atDjB1	II	335	3 to 62	Cyt.	Sis1
17	AT3G08910	atDjB4	II	323	3 to 62	Cyt.	Sis1
18	AT1G10350	atDjB5	II	349	3 to 62	Cyt.	Sis1
19	AT4G10130	atDjC13	III	174	10 to 71	Cyt.	Jjj3
20	AT1G77020	atDjC90	III	379	5 to 63	Cyt.	Djp1
21	AT2G01710	atDjC48	III	311	70 to 131	Cyt.	-
22	AT2G05230	atDjC78	III	706	65-122	Cyt.	-
23	AT2G05250	atDjC95	III	706	65-122	Cyt.	-
24	AT2G25560	atDjC44	III	656	65 to 122	Cyt.	-
25	AT2G33735	atDjC56	III	119	21 to 79	Cyt.	-
26	AT3G06778	atDjC94	III	229	41 to 98	Cyt.	-
27	AT3G62190	atDjC84	III	138	4 to 66	Cyt.	-
28	AT4G07990	atDjC88	III	230	165 to 228	Cyt.	-
29	AT4G09350	atDjC85	III	249	105 to 164	Cyt.	-
30	AT5G16650	atDjC36	III	128	10 to 68	Cyt.	-
31	AT5G37760	atDjC64	III	207	101 to 159	Cyt.	-
32	AT2G26890	atDjC30	III	2554	1523 to 1581	Cyt.	-
33	AT5G12430	atDjC34	III	1165	1023 to 1101	Cyt.	-
34	AT1G75100	atDjC18	III	651	591 to 650	Cyt.	Swa2
35	AT1G21660	atDjC10	III	523	460 to 522	Cyt.	Swa2
36	AT4G12770	atDjC9	III	891	825 to 890	Cyt.	Swa2
37	AT4G12780	atDjC5	III	904	838 to 902	Cyt.	Swa2
38	AT4G36520	atDjC8	III	1422	1357 to 1422	Cyt.	Swa2
39	AT1G30280	atDjC11	III	455	386 to 452	Cyt.	Swa2
40	AT5G06110	atDjC2	III	663	99 to 174	Cyt.	Zuo1
41	AT3G11450	atDjC1	III	647	98 to 173	Cyt.	Zuo1
42	AT1G56300	atDjC53	III	156	12 to 74	Cyt.	-

43	AT1G71000	atDjC31	III	165	7 to 69	Cyt.	-
44	AT1G72416	atDjC54	III	201	4 to 65	Cyt.	-
45	AT2G41000	atDjC80	III	211	2 to 65	Cyt.	-
46	AT3G14200	atDjC32	III	230	11 to 72	Cyt.	-
47	AT5G37750	atDjC62	III	241	69 to 126	Cyt.	-
48	AT5G62780	atDjC42	III	207	17 to 86	Cyt.	-
49	AT2G41520	atDjC72	III	1108	977 to 1054	Cyt.	-
50	AT1G79030	atDjC40	III	561	293 to 353	Cyt.	-
51	AT1G16680	atDjC39	III	554	290 to 350	Cyt.	-
52	AT1G59980	atDjB13	II	414	22 to 80	N/C	-
53	AT1G74250	atDjC12	III	630	10 to 73	N/C	Jjj1
54	AT1G75310	atDjC3	III	1448	1374 to1448	N/C	Swa2
55	AT5G23590	atDjC37	III	296	5 to 68	N/C	Cwc23
56	AT1G65280	atDjC67	III	588	304 to 361	N/C	-
57	AT1G69060	atDjC69	III	630	575 to 626	N/C	-
58	AT2G35540	atDjC52	III	590	70 to 127	N/C	-
59	AT3G04980	atDjC46	III	1165	47 to 104	N/C	-
60	AT3G06340	atDjC77	III	673	66 to 123	N/C	-
61	AT3G12170	atDjC33	III	262	10 to 68	N/C	-
62	AT4G19570	atDjC41	III	558	65 to 122	N/C	-
63	AT4G19580	atDjC66	III	312	65 to 122	N/C	-
64	AT4G19590	atDjC65	III	345	55 to 112	N/C	-
65	AT5G06910	atDjC24	III	284	28 to 86	N/C	-
66	AT5G18750	atDjC49	III	884	65 to 122	N/C	-
67	AT5G22080	atDjC35	III	246	37 to 94	N/C	-
68	AT5G27240	atDjC45	III	1104	65 to 122	N/C	-
69	AT5G37380	atDjC73	III	431	65 to 122	N/C	-
70	AT5G37440	atDjC63	III	287	73 to 130	N/C	-
71	AT5G53150	atDjC43	III	726	65 to 122	N/C	-
72	AT3G62600	atDjB18	II	346	25 to 83	ER	-
73	AT5G05750	atDjB15	II	294	113 to 170	ER	Hlj1
74	AT3G08970	atDjB9	II	572	26 to 83	ER	-
75	AT3G57340	atDjB14	II	367	112 to 169	ER	Hlj1
76	AT4G21180	atDjC21	III	661	98 to 156	ER	Sec63
77	AT1G79940	atDjC20	III	687	98 to 156	ER	Sec63
78	AT1G61770	atDjC22	III	300	33 to 90	ER	-
79	AT5G03160	atDjC19	III	482	369 to 428	ER	-
80	AT5G48030	atDjA3	I	456	93 to 151	M	Mdj1
81	AT1G28210	atDjA9	I	427	47 to 105	M	-
82	AT3G58020	atDjC71	III	254	190 to 252	M	-
83	AT1G72070	atDjC74	III	126	38 to 93	M	-
84	AT2G18465	atDjC82	III	268	206 to 268	M	-
85	AT4G37480	atDjC81	III	531	55 to 115	M	-
86	AT5G18140	atDjC79	III	333	76 to 133	M	-
87	AT5G03030	atDjC14	III	112	52 to 107	M	Mdj2
88	AT3G09700	atDjC16	III	112	52 to 106	M	Pam18

89	AT5G06410	atDjC17	III	252	92 to 157	M	Jac1
90	AT1G77930	atDjC75	III	271	75 to 139	M	-
91	AT1G80030	atDjA7	I	500	74 to 131	P	-
92	AT3G17830	atDjA4	I	517	62 to 119	P	Mdj1
93	AT2G22360	atDjA6	I	442	85 to 142	P	Mdj1
94	AT4G39960	atDjA5	I	447	84 to 141	P	Mdj1
95	AT3G17830	atDjA4	I	517	62 to 119	P	-
96	AT2G35795	atDjC15	III	112	52 to 106	P	Pam18
97	AT2G42080	atDjC70	III	263	199 to 261	P	-
98	AT3G13310	atDjC76	III	157	63 to 118	P	-
99	AT2G17880	atDjC27	III	160	67 to 127	P	-
100	AT2G42750	atDjC87	III	344	75 to 133	P	-
101	AT3G05345	atDjC93	III	244	43 to 101	P	-
102	AT4G13830	atDjC29	III	197	65 to 125	P	-
103	AT4G36040	atDjC26	III	161	64 to 125	P	-
104	AT5G23240	atDjC86	III	465	49 to 105	P	-
105	AT1G80920	atDjC25	III	163	54 to 111	P	-
106	AT5G59610	atDjC83	III	268	72 to 129	P	-

Summary of *Arabidopsis* J protein

**Localization**	**Class I**	**Class II**	**Class III**	
Cytosolic (Cyt.)	2	16	33	**51**
Nuclear/Cytosolic (N/C)	-	1	19	**20**
ER (ER)	0	4	4	**8**
Mitochondrial (M)	2	-	9	**11**
Plastid (P)	5	-	11	**16**
	**9**	**21**	**76**	**Total**

In total, 40 *A. thaliana* J proteins are placed in their orthologous context.

A. thaliana orthologs of Xdj1, Apj1, Jjj2, Scj1, Erj5, Jid1, Jem1 could not be predicted.

AT1G21080 and AT2G21510 are predicted to be orthologs of Caj1 and Djp1 showing high sequence identity with their paralogs AtDjB8 and AtDjB7. Thus their Standard names (previously suggested by Rajan and D'Silva, 2009) are changed from AtDjC92 to AtDjB19 and from AtDjC91 to AtDjB20 respectively.

AT1G28210 is a class I J protein as it has ZnF and CTD. Standard name updated from AtDjC23 to AtDjA9.

AT3G47940 is a class II J protein as it has J+G/F and CTD but lacks ZnF and is predicted to be a Sis1 ortholog. Standard name updated from AtDjC57 to AtDjB17.

AT3G62600 is a class II J protein as it has J+G/F and CTD but lacks ZnF. Standard name updated from AtDjA8 to AtDjB18.

AT3G05345 and AT3G06778 were identified as new J proteins encoding loci from TAIR database. They were assigned new standard name as AtDjC93 and AtDjC94, both belonging to class III J protein.

AT2G05250 is new J protein encoding locus identified from TAIR database showing 100% sequence identity with its paralogatDjC78. It was given new standard name as atDjC95.

Table S3. List of cytosolic J proteins orthologs from various taxa.

Sequence code	NCBI Accession no.	Sequence length (AA)	Terminal branch length value
Scerevisiae_Jjj1	NP_014172.1	590	0.352427
Cglabrata_Jjj1	KTA98196.1	623	0.316156
Calbicans_Jjj1	KHC46403.1	584	0.281206
Klacttis_Jjj1	XP_453932.1	620	0.351627
Agossypii_Jjj1	NP_982753.1	552	0.339082
Dhansenii_Jjj1	XP_015468188.1	606	0.381395
Ylipolytica_Jjj1	XP_500925.1	524	0.627792
Spombe_Jjj1	NP_593763.1	380	1.28889
Celegans_Jjj1	NP_499759.1	510	0.712284
Dmelanogaster_Jjj1	NP_611986.2	540	0.46654
Mmusculus_Jjj1	XP_017172290.1	476	0.0476507
Hsapiens_Jjj1	XP_005248306.2	553	0.0834727
Athaliana_Jjj1	OAP14759.1	630	0.329061
Osativa_Jjj1	EAZ20590.1	593	0.492738
Scerevisiae_Cwc23	EGA86912.1	283	0.865942
Cglabrata_Cwc23	XP_447661.1	296	0.85444
Calbicans_Cwc23	XP_717265.1	278	0.673229
Klacttis_Cwc23	XP_452310.1	270	1.04996
Agossypii_Cwc23	NP_986207.1	273	0.680423
Dhansenii_Cwc23	XP_460294.2	360	0.46952
Ylipolytica_Cwc23	XP_504419.1	280	0.949755
Spombe_Cwc23	NP_587857.2	289	0.735197
Celegans_Cwc23	NP_505178.1, Dnj22	296	1.08171
Dmelanogaster_Cwc23	NP_650056.1	299	0.479716
Mmusculus_Cwc23	NP_631878.2	303	0.0502456
Hsapiens_Cwc23	NP_060633.1	304	0.0538277
Athaliana_Cwc23	NP_197749.2	296	0.301913
Osativa_Cwc23	XP_015630744.1	296	0.742908
Scerevisiae_Jjj3	NP_012631.3	172	0.433602
Cglabrata_Jjj3	KTB11473.1	175	0.413928
Calbicans_Jjj3	XP_719594.1	149	0.41784
Klacttis_Jjj3	XP_452330.1	162	0.325274
Agossypii_Jjj3	NP_986186.1	156	0.699005
Dhansenii_Jjj3	XP_459949.1	168	0.362666
Ylipolytica_Jjj3	XP_503631.1	163	0.607335
Spombe_Jjj3	NP_594366.1	139	0.816392
Mmusculus_Jjj3	AAK21968.1	196	0.156442
Hsapiens_Jjj3	NP_859057.4	149	0.0550867
Athaliana_Jjj3	NP_192751.1	174	0.29945
Osativa_Jjj3_1_28590	XP_015612505.1	196	0.261188
Osativa_Jjj3_2_36980	BAF23943.1	174	0.23683
Scerevisiae_Sis1	NP_014391.1	352	0.102962
Cglabrata_Sis1	XP_446955.1	349	0.09499
Calbicans_Sis1	XP_713528.1	343	0.118659

Klactis_Sis1	XP_453274.1	354	0.115871
Agossypii_Sis1	NP_982534.1	349	0.182375
Dhansenii_Sis1	XP_462012.2	337	0.176942
Ylipolytica_Sis1	XP_503904.1	368	0.318774
Spombe_Sis1	NP_588477.1	379	0.378692
Celegans_Sis1(Dnj13)	NP_496468.1, Dnj13	331	0.391177
Dmelanogaster_Sis1_1_CG5001	NP_608586.2	350	0.343975
Dmelanogaster_Sis1_2_DNAJ1	NP_523936.2	334	0.252411
Mmusculus_Sis1_1_DNAJB1	NP_061278.1	340	0.0303552
Mmusculus_Sis1_2_DNAJB4	NP_080202.1	337	0.047458
Mmusculus_Sis1_3_DNAJB5	EDL02504.1	348	0.000194245
Hsapiens_Sis1_1_JB1	CAG38724.1	340	0.0150035
Hsapiens_Sis1_2_JB5	BAG61568.1	348	0.00789918
Hsapiens_Sis1_3_JB13	NP_705842.2	316	0.552441
Hsapiens_Sis1_4_JB4	NP_008965.2	337	0.0102739
Athaliana_Sis1_1_atDjB2	NP_194577.1	348	0.0871739
Athaliana_Sis1_2_atDjB3	NP_179646.1	337	0.0488776
Athaliana_Sis1_3_atDjB10	NP_197935.1	347	0.31175
Athaliana_Sis1_4_atDjB6	NP_197935.1	331	0.0819594
Athaliana_Sis1_5_atDjB17	NP_176181.1	350	0.303235
Athaliana_Sis1_6_atDjB1	NP_190377.1	335	0.10158
Athaliana_Sis1_7_atDjB4	NP_195759.1	323	0.0561663
Athaliana_Sis1_8_atDjB5	NP_187503.1	349	0.11179
Osativa_Sis1_1_13760_JB1	XP_015619091.1	349	0.24868
Osativa_Sis1_2_48810_JB4	XP_015637533.1	362	0.119333
Osativa_Sis1_3_20394	XP_015625804.1	349	0.120663
Osativa_Sis1_4_03630	XP_015637514.1	322	0.0357916
Scerevisiae_Swa2	NP_010606.1	668	0.461323
Cglabrata_Swa2	XP_447105.1	661	0.567029
Calbicans_Swa2	XP_714220.1	773	0.537247
Klacttis_Swa2	XP_451983.1	621	0.624043
Agossypii_Swa2	NP_987018.1	648	0.713974
Dhansenii_Swa2	XP_002770405.1	788	0.427708
Ylipolytica_Swa2	XP_500799.1	915	0.662572
Spombe_Swa2	NP_593480.1	697	0.847548
Celegans_Swa2	NP_001256947.1	441	0.665071
Dmelanogaster_Swa2	NP_649438.1	1165	0.826163
Mmusculus_Swa2	NP_001158055.1	900	0.0319171
Hsapiens_Swa2	NP_055602.1	913	0.026312
Athaliana_Swa2_1_atDjC10	NP_173585.1	523	0.608324
Athaliana_Swa2_2_atDjC9	NP_193013.2	891	0.0179154
Athaliana_Swa2_3_atDjC5	NP_193014.5	904	0.03858
Athaliana_Swa2_4_atDjC8	NP_195370.5	1422	0.771956
Athaliana_Swa2_5_atDjC3	NP_177666.1	1448	0.953884
Athaliana_Swa2_6_atDjC18	NP_565101.1	651	0.973948
Athaliana_Swa2_7_atDjC11	NP_174319.1	455	1.04475
Osativa_Swa2_1_44310	XP_015618693.1	1472	0.387281
Osativa_Swa2_2_50370	XP_015640012.1	1423	0.37916
Osativa_Swa2_3_36180	XP_015640012.1	925	0.149343
Osativa_Swa2_4_25320	XP_015620019.1	948	0.406598

Osativa_Swa2_5_43950	XP_015620955.1	888	0.251741
Scerevisiae_Ydj1	NP_014335.1	409	0.116576
Cglabrata_Ydj1	XP_448143.1	407	0.0756627
Calbicans_Ydj1	XP_717458.1	393	0.250831
Klactis_Ydj1	XP_455231.1	409	0.132644
Agossypii_Ydj1	NP_983839.1	410	0.142997
Dhansenii_Ydj1	XP_461267.1	406	0.162755
Ylipolytica_Ydj1	XP_504839.1	417	0.365534
Spombe_Ydj1	NP_595428.1	407	0.363966
Celegans_Ydj1_1	NP_493570.1	402	0.485477
Celegans_Ydj1_2	NP_504452.1	439	0.594633
Dmelanogaster_Ydj1_1	NP_650283.1	403	0.32156
Dmelanogaster_Ydj1_2	AAL68031.1	389	0.928638
Mmusculus_Ydj1_1	XP_006511388.1	397	0.179872
Mmusculus_Ydj1_2	NP_032324.1	397	0.00477068
Mmusculus_Ydj1_3	NP_062768.1	412	0.00454273
Hsapiens_Ydj1_1_DnaJA1	NP_001530.1	397	0.000000005
Hsapiens_Ydj1_2_DnaJA2	NP_005871.1	412	0.000000005
Athaliana_Ydj1_1_AtDjA2	NP_568412.1	419	0.058041
Athaliana_Ydj1_2_AtDjA1	NP_189997.1	420	0.022253
Osativa_Ydj1_1_44620	XP_015630926.1	417	0.0622473
Osativa_Ydj1_2_57340	XP_015632121.1	417	0.061877
Osativa_Ydj1_3_43930	XP_015626316.1	421	0.0818759
Osativa_Ydj1_4_46390	XP_015635751.1	416	0.112989

Table S4. Statistical analysis of evolutionary rate of J protein orthologous group.

Calculation of mean branch lengths for evolutionary rate prediction of different J proteins

	Ydj1	Sis1	Jjj3	Cwc23	Jjj1
**No. of sequences**	23	30	13	14	14
**Sum of branch Lengths**	4.53	4.767	5.085	8.989	6.07
**Mean branch length**	0.1969	0.1589	0.3912	0.6421	0.4336
**Std. Error**	0.04745	0.02552	0.05925	0.08892	0.08106

One-way analysis of variance

**P value**	**<0.0001**		
**P value summary**	***		
Are means signif. different? (P < 0.05)	Yes		
Number of groups	6		
F	11.28		
R squared	0.3348		
Bartlett's test for equal variances			
Bartlett's statistic (corrected)	20.7		
P value	0.0009		
P value summary	***		
Do the variances differ signif. (P < 0.05)	Yes		
ANOVA Table	SS	df	MS
Treatment (between columns)	3.604	5	0.7208
Residual (within columns)	7.16	112	0.06393
Total	10.76	117	

Mann Whitney test results

**Ydj1 vs Sis1**		**Ydj1/Sis1 vs all Class III**	
Mann Whitney test		Unpaired t test	
P value	0.9356	P value	< 0.0001
P value summary	ns	P value summary	***
Are medians signif. different? (P < 0.05)	No	Are means signif. different? (P < 0.05)	Yes
One- or two-tailed P value?	Two-tailed	One- or two-tailed P value?	Two-tailed
Mann-Whitney U	340	t, df	t=6.821 df=116

Ydj1/Sis1 vs Jjj3

Mann Whitney test	
P value	0.0007
P value summary	***
Are medians signif. different? (P < 0.05)	Yes
One- or two-tailed P value?	Two-tailed
Mann-Whitney U	134

Ydj1/Sis1 vs Swa2

Mann Whitney test	
P value	< 0.0001
P value summary	***
Are medians signif. different? (P < 0.05)	Yes
One- or two-tailed P value?	Two-tailed
Mann-Whitney U	243

Ydj1/Sis1 vs Jjj1

Mann Whitney test	
P value	0.0005
P value summary	***
Are medians signif. different? (P < 0.05)	Yes
One- or two-tailed P value?	Two-tailed
Mann-Whitney U	146

Ydj1/Sis1 vs Cwc23

Mann Whitney test	
P value	< 0.0001
P value summary	***
Are medians signif. different? (P < 0.05)	Yes
One- or two-tailed P value?	Two-tailed
Mann-Whitney U	107

Cwc23 vs Jjj1

Mann Whitney test	
P value	0.0565
P value summary	ns
Are medians signif. different? (P < 0.05)	No
One- or two-tailed P value?	Two-tailed
Mann-Whitney U	56

**Cwc23 vs Swa2**	**Cwc23 vs Swa2**
Mann Whitney test	
P value	0.1422
P value summary	ns
Are medians signif. different? (P < 0.05)	No
One- or two-tailed P value?	Two-tailed
Mann-Whitney U	119

Cwc23 vs Jjj3

Mann Whitney test	
P value	0.0308
P value summary	*
Are medians signif. different? (P < 0.05)	Yes
One- or two-tailed P value?	Two-tailed
Mann-Whitney U	46

Method S1 Sequence analysis of *Arabidopsis thaliana* J protein

Multiple loci were previously predicted to code for J proteins in *A. thaliana*: At1G80120, At1G69050 At5G34895, At2G07010, At2G14930, At2G13940, At2G24660, At1G31210, and At2G02200. However, the proteins encoded by these loci do not contain a predicted J domain and were thus removed from the list. Protein accession number BAA97287 was also excluded from our list for the same reason. Similarly gene locus F1B16.14 had only protein sequence information in the NCBI database with accession number AAG13076 but has no data on TAIR and appeared to be a splice variant of J protein locus At1G75310. Loci At1G79030 and At1G56300 were included twice with different names in the previous list, thus considered as one in our refined list of J proteins (S1 Table). Loci At5G11500, CAB91598, At3G59280, and At5G61880 were predicted to be J-like proteins (JLP) and thus are not included in our list of J proteins. JLPs have a domain similar to J domain but the critical HPD motif is not present making them non-functional in Hsp70-dependent functions.
